# Stay in shape: Assessing the adaptive potential of shell morphology and its sensitivity to temperature in the invasive New Zealand mud snail *Potamopyrgus antipodarum* through phenotypic plasticity and natural selection in Europe

**DOI:** 10.1002/ece3.9314

**Published:** 2022-10-01

**Authors:** Lisa Männer, Carolin Mundinger, Martin Haase

**Affiliations:** ^1^ AG Vogelwarte, Zoological Institute and Museum University of Greifswald Greifswald Germany; ^2^ AG Applied Zoology and Nature Conservation, Zoological Institute and Museum University of Greifswald Greifswald Germany

**Keywords:** climate change, clonal reproduction, common garden experiment, genetic adaptation, geometric morphometrics, heritability

## Abstract

Climate change may force organisms to adapt genetically or plastically to new environmental conditions. Invasive species show remarkable potential for rapid adaptation. The ovoviviparous New Zealand mud snail (NZMS), *Potamopyrgus antipodarum*, has successfully established across Europe with two clonally reproducing mitochondrial lineages since its arrival in the first half of the 19th century. Its remarkable variation in shell morphology was shown to be fitness relevant. We investigated the effects of temperature on shell morphology across 11 populations from Germany and the Iberian Peninsula in a common garden across three temperatures. We analyzed size and shape using geometric morphometrics. For both, we compared reaction norms and estimated heritabilities. For size, the interaction of temperature and haplotype explained about 50% of the total variance. We also observed more genotype by environment interactions indicating a higher degree of population differentiation than in shape. Across the three temperatures, size followed the expectations of the temperature‐size rule, with individuals growing larger in cold environments. Changes in shape may have compensated for changes in size affecting space for brooding embryos. Heritability estimates were relatively high. As indicated by the very low coefficients of variation for clonal repeatability (*CV*_*A*_), they can probably not be compared in absolute terms. However, they showed some sensitivity to temperature, in haplotype t more so than in z, which was only found in Portugal. The low *CV*_*A*_ values indicate that genetic variation among European populations is still restricted with a low potential to react to selection. A considerable fraction of the genetic variation was due to differences between the clonal lineages. The NZMS has apparently not been long enough in Europe to accumulate significant genetic variation relevant for morphological adaptation. As temperature is obviously not the sole factor influencing shell morphology, their interaction will probably not be a factor limiting population persistence under a warming climate in Europe.

## Graphical abstract

In invasive populations of the New Zealand Mud Snail in Europe shell morphology, which is fitness relevant, is sensitive to temperature. The genetic variation underlying size and shape is very limited. As temperature is obviously not the sole factor influencing shell morphology, their interaction will probably not be a factor limiting population persistence under a warming climate in Europe.

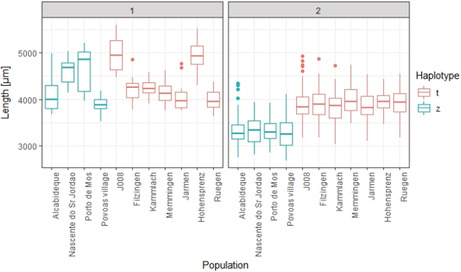

## 1 INTRODUCTION

The increasingly visible effects of anthropogenic climate change have a growing impact on the organisms on the planet (Magnan et al., 2021; Malhi et al., 2020; Soravia et al., 2021; van der Heide et al., 2021). The effects organisms have to deal with include, among others, temperature rise, flooding events increasing in number and intensity, and sea level rise (Masson‐Delmotte et al., 2021). Different species have different ways to react to those environmental changes (Harmon & Barton, 2013). Some species may not be able to cope with the new climate conditions and die out while others will be able to shift their distribution to suitable sites. Others have the capability to respond to new environmental conditions via genetic adaptation or phenotypic plasticity (Barbet‐Massin et al., 2012; Bellard et al., 2012; Gienapp et al., 2008; Salamin et al., 2010; Thomas et al., 2004). In order to understand and predict potential reactions and eventually develop appropriate conservation measures, it is of utmost importance to study as many species as possible with respect to their adaptability and potential to successfully disperse. Particularly well‐suited models to study short‐term responses to novel habitats are invasive species immigrating into new, non‐native habitats and reproducing successfully. In many cases, a high capacity for phenotypic plasticity is an important reason for the success of invasive species (Davidson et al., 2011). Additionally, some invasive species reproduce asexually and thus show higher reproductive rates (Frankham, 2005; Mergeay et al., 2006; Sakai et al., 2001; Xie et al., 2010).

A phenotypically plastic genotype has the ability to produce more than one phenotype when exposed to different environments and, therefore, has a higher chance of survival across a range of environments (Price et al., 2003). Genetic adaptation is the result of natural selection of beneficial alleles or genotypes and important factors for genetic variability in a population are mutation, migration, genetic drift, and recombination (Carja et al., 2014; Star & Spencer, 2013). Founder events, the establishment of new populations from just a small number of individuals originating from a large ancestral population, suggest that invasive species have a reduced genetic variability when invading new localities, as they carry only a fraction of the total genetic variation (Lee, 2002). The phenomenon that invasive species successfully invade new environments despite having a low genetic variance is called the “Genetic Paradox of Biological Invasion” (Estoup et al., 2016; Frankham, 2005; Kolbe et al., 2004). However, various studies found that not all invasive species show a lower genetic variability and that diversity differs a lot between species (Bossdorf et al., 2005; Dlugosch & Parker, 2008; Kolbe et al., 2004; Lee, 2002; terHorst et al., 2018).

The New Zealand mud snail (NZMS) *Potamopyrgus antipodarum* is a particularly suitable invasive species to investigate genetic adaptation and phenotypic plasticity. This fresh and brackish water snail originates from New Zealand and was introduced to Australia, Europe, North America, Japan, Chile, and most recently reached northern Africa (Alonso & Castro‐Díez, 2012; Collado, 2014; Taybi et al., 2021). The NZMS had already been introduced to Europe by the mid‐19th century, probably in the ballast water of ships (Ponder, 1988; Smith, 1889). Being ovoviviparous, *P. antipodarum* reproduces both sexually and asexually in its native range. The sexually reproducing males and females are diploid, the asexually reproducing females di‐, tri‐, or tetraploid. As asexually reproducing females produce offspring by apomictic parthenogenesis, all offspring are identical clones of their mother (Dybdahl & Lively, 1995; Liu et al., 2012; Lively, 1987; Neiman et al., 2011; Paczesniak et al., 2013; Phillips & Lambert, 1989; Wallace, 1992). In invaded habitats, however, female individuals of *P. antipodarum* reproduce only asexually (Duft et al., 2007; Ponder, 1988). Consequently, the genetic variation in Europe is low: based on minisatellites, Hauser et al. (1992) identified 3 genotypes occurring in the UK (66 individuals from a total of 11 populations). Jacobsen et al. (1996) detected two clonal lineages among Danish populations using RAPDs (117 individuals from 10 populations). Two mitochondrial haplotypes were found across several countries (Butkus et al., 2020; Städler et al., 2005; Verhaegen, McElroy, et al., 2018). Among these two haplotypes, Verhaegen, McElroy, et al. (2018) identified 10 SNP genotypes (425 individuals from 21 localities).

The apparent adaptability and the rapid population growth make this snail one of the most concerning alien species in Europe (Alonso & Castro‐Diez, 2008; Nentwig et al., 2018). The adaptability comprises various traits including shell morphology (Haase, 2003; Kistner & Dybdahl, 2014; Verhaegen, McElroy, et al., 2018; Verhaegen, Neiman, et al., 2018). Shape and size in different environments may be related to fitness through their effects on brood size, as in the absence of flow, larger and wider snails carry more embryos. Considering flow (and other parameters), the interaction of morphology and fitness becomes more complex (Verhaegen et al., 2019; Verhaegen, McElroy, et al., 2018; Verhaegen, Neiman, et al., 2018). Shell morphology may not only be related to brood size but also to survival providing crush resistance after dislodgement or against predators (Holomuzki & Biggs, 2006; Verhaegen et al., 2019). In its native range, the NZMS exhibits high genetic and morphological variation, but as clonal lineages keep evolving repeatedly (Neiman et al., 2005; Paczesniak et al., 2013), there is no difference in the morphological variation between sexually and asexually reproducing snails (Verhaegen, Neiman, et al., 2018). In Europe (and other invaded areas), in contrast to the native range, both genetic and morphological variation are reduced as a consequence of the founder effect. Field studies suggested that phenotypic plasticity is an important driver for shaping the reduced but still observed morphological variation in Europe (Verhaegen, McElroy, et al., 2018). However, the potential for genetic adaptation of invasive populations is largely unexplored. Such investigations require lab‐controlled common garden experiments (Dybdahl & Kane, 2005; Kistner & Dybdahl, 2013).

In this study, we investigated the effects of temperature on shell morphology and reproduction. The temperature‐size rule predicts delayed sexual maturation at larger body sizes at lower temperatures in ectotherm invertebrates (Angilletta et al., 2004; Atkinson, 1994, 1995). This has also been shown for North American populations of the NZMS (Dybdahl & Kane, 2005). Whether these populations are related to the European ones cannot be told in retrospect as the NZMS has apparently been introduced to the Western US twice independently from Europe and New Zealand (Donne et al., 2020). The shell shape has been suggested to play a role in temperature regulation with more globular shells developing at lower temperatures (Albarrán‐Mélzer et al., 2020; Wong & Lim, 2017). In order to disentangle environmental from genetic effects, we kept asexually reproducing snails from 11 European populations in climate cabinets at three different temperatures to produce offspring. We recorded how long it took until the required number of offspring was produced. Morphology of fully grown shells (Verhaegen, McElroy, et al., 2018) was analyzed in the framework of geometric morphometrics. We were particularly interested in the amount of genetic variation we might possibly detect among the populations. As *P. antipodarum* has only been present in Europe for around 180 to 360 generations and populations were founded by probably only few individuals (Donne et al., 2020; Ponder, 1988; Verhaegen et al., 2021), our results may shed light on the process of differentiation in this clonal invader, hence its adaptive potential. As the genetic variation in Europe is certainly still low, we had the following null expectations: (1) shells of snails from different populations would become more similar under the same environmental conditions; (2) the slopes of the reaction norms would be similar across the three temperatures; (3) that the variance of the phenotypic traits could be largely explained by environmental effects, hence broad‐sense heritability would be low and (4) that individuals that were exposed to lower temperatures would develop larger and more globular shells and have lower reproductive rates. Deviations from expectations 1–3 would indicate genetic differentiation among populations.

## 2 MATERIAL AND METHODS

### 2.1 Sample collection

**FIGURE 1:**
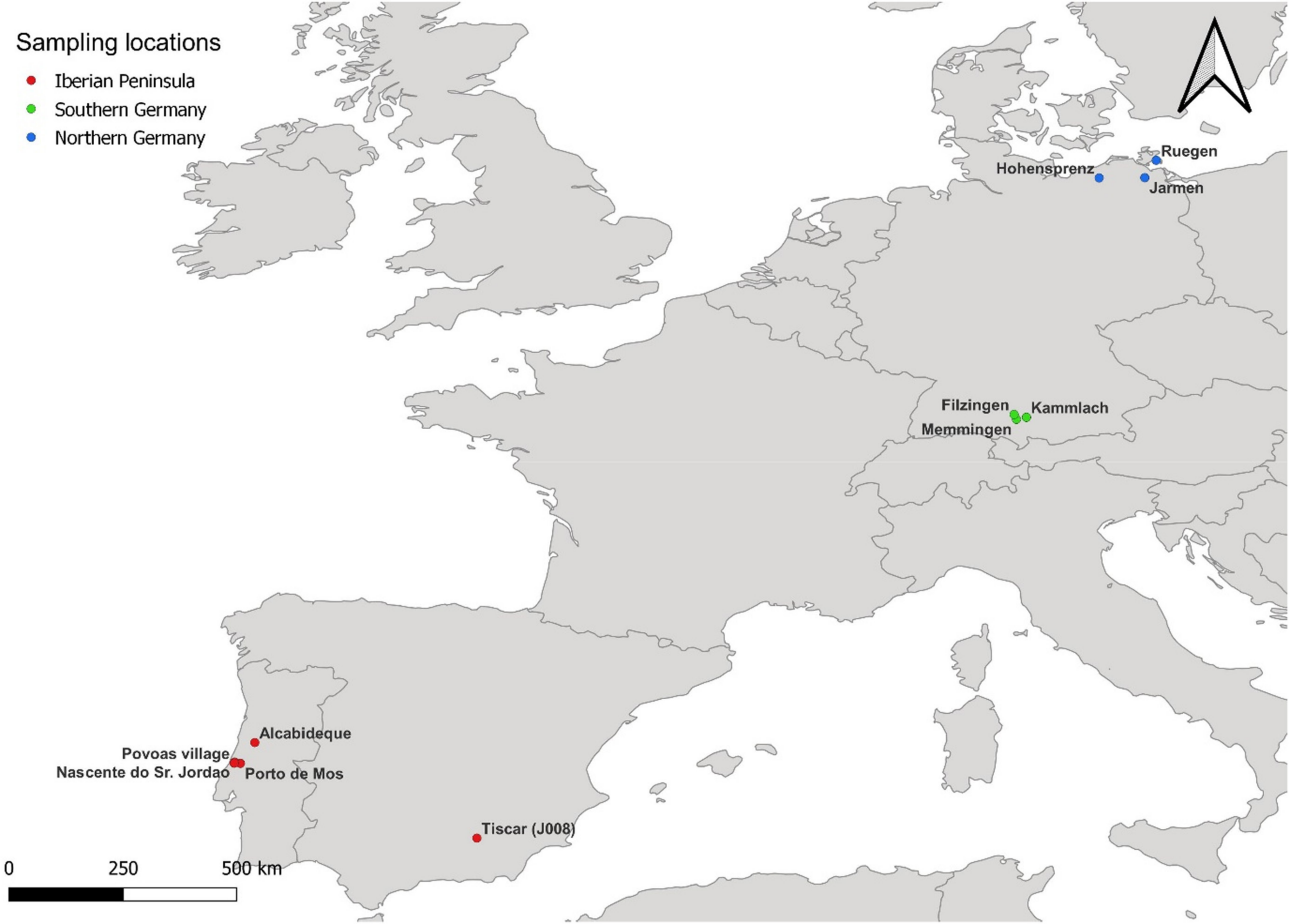
Collection sites along the northeast‐southwest gradient within Europe.

For the experiment, we used snails along a northeast‐southwest gradient within Europe: three populations each from Northern and Southern Germany (six total), one Spanish population, and four Portuguese populations (Figure 1). The Spanish and the Portuguese populations were joined in the region “Iberian Peninsula” in our analysis. Collection dates, localities, coordinates, habitat types, and the water temperature at the collection date of all samples are given in Table 1. Both mitochondrial haplotypes were represented in our samples (see Section 3). Snails with haplotype t commonly live in freshwater, while snails possessing haplotype z are found in brackish water. Populations with snails of both haplotypes are rare and have only been found near the coast (Butkus et al., 2020; Verhaegen, McElroy, et al., 2018). Although these two lineages normally occur in different salinities, all snails were collected in freshwater habitats (salinities: 0.146‰–0.404‰) using a small fishing net and a white tray for sorting the animals among the sand and gravel taken with the net. Living snails were transported to the Greifswald laboratory in small plastic jars filled with water from the collecting site. A smaller number of individuals were fixed immediately in ethanol (96%, MEK). The living snails were subsequently kept in a climate cabinet at 19°C in small 700 ml aquariums until the start of the experiment. Snails were fed weekly with Spirulina flakes (JBL Spirulina Premium). The water was also changed once a week.

**TABLE 1:** Locality details of the 11 populations used in the common garden experiment

Population	Region	Collection date/collection span	Coordinates	Type of water body	Temperature at collection [C°]
Hohensprenz	Northern Germany	02.07.2018	53°55′25.428″N 12°11′57.354″E	Stream	23.2
Jarmen	29.06.2018	53°55′44.952″N 13°19′0.540″E	Lake	21.5
Rügen	29.06.2018	54°21′19.421″N 13°35′52.402″E	Stream	15.7
Filzingen	Southern Germany	15.05.2018	48°8′7.697″N 10°6′55.371″E	Lake	17.8
Kammlach	17.05.2018	48°3′57.483″N 10°25′3.568″E	Stream	14.2
Memmingen	14.05.2018	48°1′3.165″N 10°10′19.766″E	Small river	12.5
J008 (Spain)	Iberian Peninsula	14.07.2018	37°46′05.0″N 3°01′26.8″W	Trough	–
Alcabideque	20.06.2018	40°6′23.311″N 8°27′54.130″W	Lake	17.0
Nascente do Sr. Jordão	21.06.2018	39°37′41.958″N 8°57′29.599″W	Stream	20.1
Porto de Mós	21.06.2018	39°35′46.049″N 8°49′2.906″W	Small river	24.0
Póvoas village	22.06.2018	39°36′7.236″N 8°58′12.964″W	Spring	17.8

### 2.2 Common garden experiment

At the beginning of the experiment, six mother snails per population were placed individually in small glass jars with 3 ml of sand (JBL Sansibar, Red) to cover the bottom and 250 ml of artificial fresh water (salt content: 0.5‰). A salinity of 0.5‰ ranges at the upper end of the salinities measured at the sampling sites. Additionally, we placed a small stone for cover and some pieces of shell of the marine bivalve *Arenomya arenaria* as a source of chalk for the snail in every jar. Stones and shell pieces were sterilized in boiling water beforehand. Snails were fed weekly with Spirulina flakes (JBL Spirulina Premium). The water was initially changed once a week, but with increasing logistic effort, the interval was increased to every 10 days. To ease the handling of the jars, we always grouped 10 of them in opaque trays, which also prevented light entering from below. After maintenance, we rotated the jars within the trays and the trays within the corresponding climate cabinet to exclude an influence on the position of the jars within the climate cabinet on the snails.

In order to investigate the reaction of shell morphology to temperature, we performed a common garden experiment (de Villemereuil et al., 2016; Moloney et al., 2009). We reared the snails in climate cabinets at three different temperatures (15, 19, and 23°C) with an artificial day‐night rhythm of 16 h day and 8 h night. These temperatures reflect temperatures measured in the field during the warmer seasons, the peaks of NZMS reproduction (Verhaegen et al., 2021). Higher temperatures are tolerated but may interrupt or slow down reproduction (Dybdahl & Kane, 2005). Feeding, maintenance, and checking for offspring fell into day hours to not disturb the darkness of the night. From each of the 11 populations, six adult mother snails per temperature were allowed to sire offspring (clones). The shells of NZMS do not change anymore once adulthood is reached (Verhaegen, McElroy, et al., 2018). As the mother snails were already adults when we placed them into the common garden experiment, we measured and analyzed their size and shape with regard to the developmental temperature conditions of their natural habitats. By contrast, the developmental temperature of the daughter generation was the temperature of the corresponding climate cabinets they were born and raised in. To avoid confusion between mother and matured daughter snails, we marked the mother with a dot of nail polish on the shell and allowed it to continue to reproduce. Once enough offspring (~10 offspring) were available, the mother snail was fixed in ethanol. We tried to limit the number of offspring per jar to 15. Apart from that, we were not able to control further for density in the jars, as some offspring died before reaching adulthood. Once an offspring had matured, it was also fixed in ethanol—except for some F1 daughter snails, which were supposed to generate an F2 daughter generation. However, as it became obvious that some F1 snails reproduced too slowly to reach a meaningful sample size within the projected experimental time, we decided to terminate this second phase of the experiment in January 2020 and focus our analyses on the F1 generation. At this point, only two Portuguese populations had produced reasonable numbers of F2‐offspring across all three temperatures. Those not adult at that time were allowed to finish growth. We fixed the last snail on June 9, 2020, 2 years after the start of the common garden experiment. These Portuguese F2 snails were additionally analyzed, and the results are reported in Appendix S7.

As *P. antipodarum* releases hatchlings consecutively over weeks and not simultaneously, a split‐brood design is generally difficult to implement and impossible at our scale. Since the asexually reproducing invasive populations in Europe are genetically very homogeneous (Butkus et al., 2020; Verhaegen, McElroy, et al., 2018; Weetman et al., 2002), our approach should still be unproblematic (Hurlbert, 1984). Compared with the number of populations, we kept the number of initial parental snails rather low, because previous experiments with North American populations suggested that differences between populations would be larger than the variation within populations (Dybdahl & Kane, 2005; Kistner & Dybdahl, 2014).

### 2.3 Morphometrics

**FIGURE 2:**
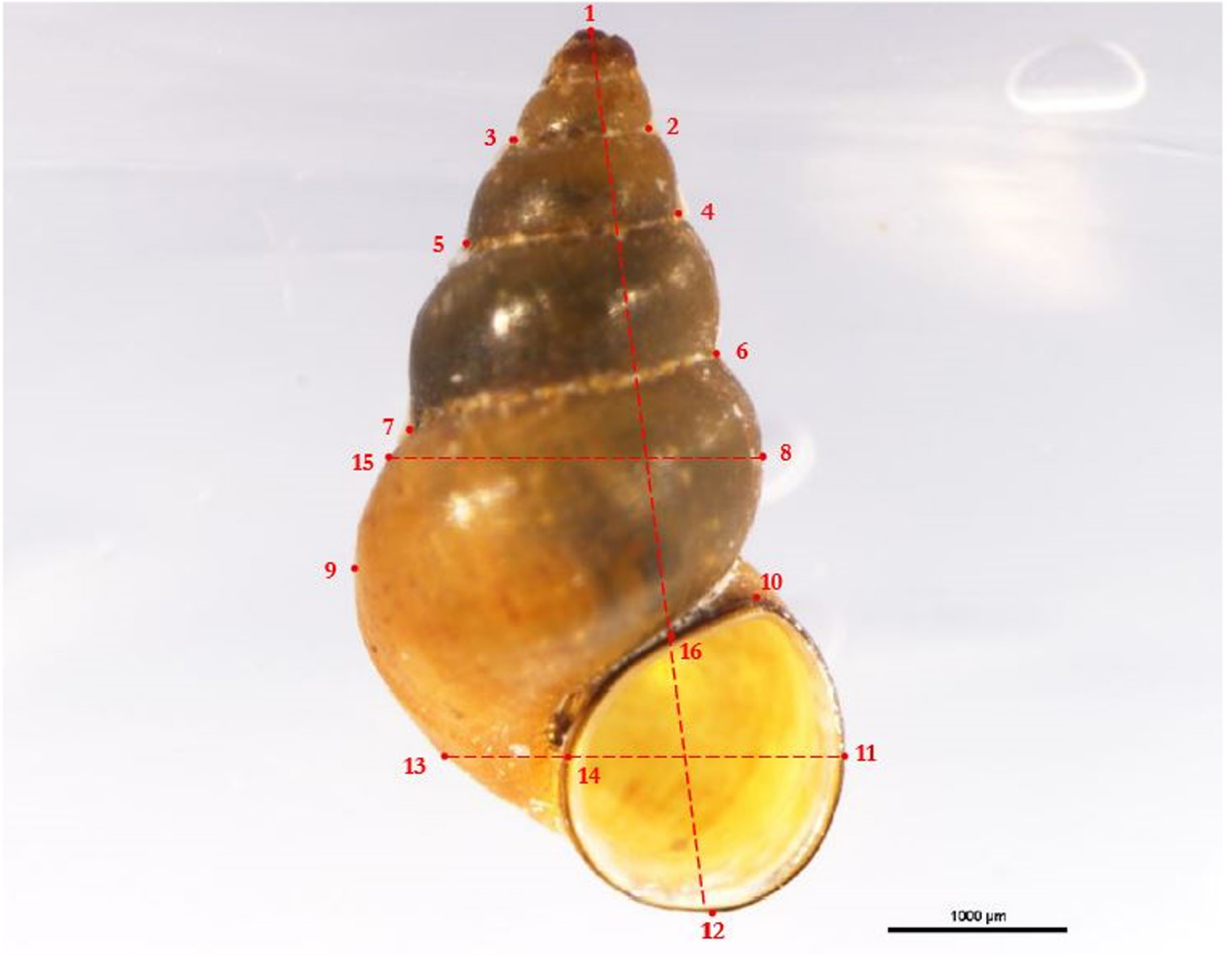
Position of landmarks. Dashed auxiliary lines indicate how landmarks 13–16 were constructed.

Photos of the fixed snails were taken with a Nikon SMZ25 stereomicroscope equipped with a Nikon DS‐Ri2 camera (Nikon, Tokio, Japan). Each snail was placed in a petri dish with silicone inlay with the aperture of the shell facing upwards and the coiling axis oriented horizontally as seen in Figure 2. Shell length was measured parallel to the coiling axis using the NIS‐Elements Ar 4.51 imaging software (Nikon, Tokio, Japan).

The shape of the shells was quantified using the geometric morphometrics approach. In contrast to traditional morphometrics based on measurements and their ratios, this method is able to quantify shape without the influence of size, which, in addition to position and rotation, is removed by the Procrustes superimposition as part of the procedure (Tatsuta et al., 2018). We transformed the photos of the shells into TPS files and placed 16 landmarks onto each shell using the programs tpsUtil64 version 1.78 and tpsDig version 2.31 (https://life.bio.sunysb.edu/morph/, both downloaded 1st of March 2021). 43 snails with damaged shells or shells covered with algae preventing unambiguous placement of landmarks were excluded. The repeatability of the entire procedure (see below for details) was tested with a set of 20 shells photographed twice at an interval of 2 weeks. All morphometric data were collected exclusively by LM in order to avoid the inflation of variance due to differences in handling by different workers (Schilthuizen & Haase, 2010). The repeatability of size measurements was statistically tested with a paired two‐sample Wilcoxon test, V(19) = 147, *p* = .12. The repeatability of the shape measurements was verified using the Integrated Morphometrics Package, TwoGroup 8: Goodall's *F* = 0.70, *p* = .91 (Sheets, 2014). For both size and shape, repeatability tests did not show any significant difference, confirming the robustness of the procedure.

For geometric morphometric analyses, we used the Integrated Morphometrics Package. CoordGen 8 was used to perform Procrustes superimposition, calculate centroid sizes, the size measure of the geometric morphometrics framework, and conduct quick diagnostic principal component analyses (PCA)—the latter as a quality control a posteriori, i.e., after setting the landmarks—through identification of outliers in the resulting scatter plot of the first two principal components (PCs). Photographs of outliers were checked again for any damage or algae on the shells, which could have misled the positioning of landmarks in tpsDig. If an outlier was deemed problematic due to such artifacts, it was eliminated from further analyses, otherwise retained. We then tested our dataset for allometry, which is the consequence of distinct growth rates of different body parts resulting in a nonlinear relationship between size and shape (Huxley & Teissier, 1936; Nakagawa et al., 2017; Outomuro & Johansson, 2017). Data were standardized accordingly with Regress 8 and all subsequent PCAs were conducted with both datasets, uncorrected and corrected, in PCAGen8. Thus, we compared the effects on shell shape as it is exposed to the environment (uncorrected) to those on theoretically pure shape (corrected; Outomuro & Johansson, 2017). We performed our analyses on the first three PCs (in the geometric morphometrics framework correctly relative warps), which depending on the dataset explained between 53.8% and 66.8% of the total variance (Table S1). As we did not observe relevant differences between the analyses of uncorrected and standardized PCs, we here report only the results for the original PCs.

Changes in shape along the PCs were visualized by deformation grids created in PCAGen8. Deformation grids show deformation from specimens with lowest to those with highest scores of variations.

### 2.4 Life history trait

The reproductive rate of the parental snails was estimated by counting the days each mother snail needed to sire ideally 10 offspring. At this point, mother snails were fixed in ethanol. Some mother snails were not able to reproduce 10 offspring until the end of the experiment, others died beforehand. We only included mother snails in our life history trait analysis that (1) produced seven or more offspring or (2) three to six offspring if they survived at least 18 months in the experiment. The influence of population, temperature of the climate cabinets, water temperature difference between habitat at the time of collection and climate cabinet, morphology, and haplotype on the reproductive rate was tested using generalized linear models (GLMs). As the habitat temperature at the time of collection of the Spanish population was not measured, we excluded the Spanish mother snails from the analysis of the reproductive rate.

### 2.5 16S rRNA sequencing

Mitochondrial lineages (used synonymously with haplotype in the course of the paper) were determined by sequencing a fragment (~500 bp) of 16S rRNA. For the snails from northern Germany, this information was taken from Verhaegen, McElroy, et al., 2018. From all other populations, we sequenced each of three already photographed individuals. Investigating only a subsample is justified as in Europe both haplotypes t and z only rarely occur in sympatry (Butkus et al., 2020; Verhaegen, McElroy, et al., 2018). DNA was extracted with the E.Z.N.A® Mollusk DNA Kit (Omega Bio‐Tek Inc.) by crushing the entire snail and following the manufacturer's protocol. The Polymerase chain reactions (PCR) were performed in a total volume of 10 μl and consisted of 1 μl of DNA solution (~20 ng), 3 μl of water, 5 μl of HS MyTaqTM RedMix (Bioline), 0.60 μl of 1% BSA and 0.20 μl of each primer (from a 10 pmol stock solution) 16Sar‐L and 16Sbr‐H (Palumbi, 1991).

The PCR temperature profile was a touch‐down protocol with 1 min of initial denaturation at 95°C, 10 cycles with 20 s of denaturation at 95°C, and 20 s of annealing starting at 60°C and dropping by 1°C in each cycle to 51°C and 30 s of extension at 72°C, followed by further 25 cycles consisting of 20 s denaturation at 95°C, 20 s of annealing at 51°C, and 30 s extension at 72°C, and a 5 min final extension at 72°C. The PCR products were visualized on a 1% agarose gel and purified with an exonuclease I and shrimp alkaline phosphatase mix.

Cycle sequencing was conducted using the BigDye™ Terminator ver. 3.1 Cycle Sequencing Kit (Applied Biosystems) with 50% replaced by halfBD (Sigma‐Aldrich) and the PCR primers. The cycle sequencing products were cleaned with magnetic beads using the HighPrepTM DTR Dye Terminator Removal Clean Up (MagBio Genomics) and then sequenced on an ABI 3130xl Genetic Analyzer (Applied Biosystems). Sequences were compiled and proof‐read with Geneious v.R10.2 (www.geneious.com) and BioEdit 7.0.5.3 (Hall, 1999).

### 2.6 Statistical analysis

All analyses were performed in R (R Core Team, 2021). To visualize the data, we created plots using the ggplot package (Hadley, 2016), for statistical tests, we used lme4, lmerTest, lmtest, and tidyverse (Bates et al., 2015; Kuznetsova et al., 2017; Wickham et al., 2019; Zeileis & Hothorn, 2002).

To analyze differences in shell size, shell shape, and reproductive rates of mother snails among and between the generations, haplotypes, and populations, we applied nonparametric Kruskal–Wallis tests followed by the Dunn's test (adjusted for multiple testing using the “BH” method by Benjamini & Hochberg, 1995), as our design was unbalanced and data showed unequal variances.

During data inspection, we noticed that snails of the parental generation were not randomly distributed according to size in the different temperature treatments. Mother snails selected for the 19°C climate cabinet were significantly smaller compared with mother snails of the 15°C treatment [Kruskal–Wallis chi‐squared = 8.7404, *df* = 2, *p* = .01265 (length); Kruskal–Wallis chi‐squared = 7.1217, *df* = 2, *p* = .02842 (centroid size)]. To correct this bias, we removed the smallest six mother snails of the 19°C treatment and all their offspring from the size analysis. After this, no significant difference in length between the different mother snails and climate cabinets remained. However, the six mother snails and their offspring stayed in the analysis for shape because we did not find any significant differences in shape across cabinets.

We further tested for multicollinearity between the different explanatory variables (temperature, region, population, and haplotype) for all of the response variables (size and shape) and model types (generalized linear models and mixed‐effect models) using the package “performance: Assessment of Regression Models Performance” (Lüdecke et al., 2021). This revealed in all cases a high collinearity for population and region (VIF > 10). Applying likelihood ratio tests (LRTs), we found that models including the population level as a fixed factor had a significantly better fit compared with models containing region. Consequently, we rejected the factor region and retained only the population as an explanatory variable for further modeling. In addition, we investigated correlations between the two size parameters, length and centroid size, and found them highly correlated (*p* < .001, *R* = .82). To avoid redundancy, we will from now on only report and show the results for the snails´ length unless centroid size shows a different outcome than length. The statistical analyses and data visualization for the centroid size can be found in Appendix S2.

To analyze the impact on the life history traits, size, and shape of the parental generation, we fit generalized linear models (GLMs), which do not require normally distributed errors of the response variables. For the morphological traits, these models included temperature, population, and haplotype as fixed factors, while for the life history traits also temperature difference, size, and shape measurements were added. To assess the impact on size and shape and the slopes of the reaction norms of the offspring generation we fit linear mixed‐effect models that allow the addition of random factors. We added the mother ID as a random factor to control for maternal effects. We then used the Akaike information criterion (AIC) to compare models and aimed for model parsimony, when AIC values did not differ by >2. Temperature in all models was treated as a categorical variable instead of a continuous one, to facilitate interpretations of two and three‐way interactions, as well as to account for potential nonlinear curves (Mazé‐Guilmo et al., 2016). We visualized the fixed variables effect of the respective best models using the “predictorEffects” function of the effects package (Fox, 2003). To quantify the explained variation in both fixed and random effects (= conditional *R* ^*2*^) and in the fixed effects only (= marginal *R* ^*2*^) (Johnson, 2014; Nakagawa & Schielzeth, 2013), we used the “r.squaredGLMM” function of the MuMIn version 1.43.17 package (Bartoń, 2021). Subtracting the marginal *R* ^*2*^ from the conditional *R* ^*2*^ yields the explained variance of the random factor of the model, which in our case was always “maternal ID,” the potential influence of the mother snails, and the conditions in the respective jars (Section 4). We built reaction slopes using the mixed ANOVA with a temperature*population interaction to analyze the treatment × genotype interactions, then calculated slopes using the “lstrends”—function of the “emmeans” R package. In the next step, we performed a pairwise comparison of all slopes, using the “pairs” functions, to calculate significant differences and directional trends of the slopes (Lenth, 2016).

### 2.7 Heritability

Heritability expresses the proportion of the total phenotypic variance of a trait, which is due to genetic variation. In this general form, we speak of broad‐sense heritability (*H* ^2^). Narrow‐sense heritability (*h* ^2^) captures the additive genetic variance, i.e., the sum of the effects of the alleles affecting a trait from both parents (Visscher et al., 2008). As our study organism, the NZMS, reproduces clonally, the additive genetic variance cannot be determined. Instead, we calculated the broad‐sense heritability with the “clonal repeatability” replacing the additive genetic variance based on an R script of Fischer et al. (2021). Additionally, we estimated the coefficient of genetic variation (*CV*_*A*_) and the evolvability, *I*_*A*_ of only the size parameter length and centroid size. In comparison to heritability, the *CV*_*A*_ measures the genetic variation standardized by the trait mean, which makes it unbiased of other causes of variation. Thus, it is an appropriate parameter for comparing studies of genetic variance (Garcia‐Gonzalez et al., 2012). *I*_*A*_ represents the expected proportional response to a unit strength of selection, hence is a suitable measure of evolvability (Hansen et al., 2003, 2011; Hereford et al., 2004). These parameters could not meaningfully be calculated for the three principal components as they are centered around zero resulting in noncomparable, large values.

To calculate the broad‐sense heritability *H* ^2^ of all size and shape parameters and the *CV*_*A*_ and the *I*_*A*_ of shell length and centroid size of the F1 generation at each temperature, we used linear mixed models implemented in the linear mixed models R package, lmerTest, using its functions “lmer” and “VarCorr” (Kuznetsova et al., 2017). According to Visscher et al. (2008), we calculated the broad‐sense heritability with the following formula:

$$H^{2}=\frac{V_{A}}{V_{A}+V_{\mathrm{res}}}$$

with *V*_*A*_ being the overall genetic variance and *V*_*res*_ the variance within the clones. The coefficient of genetic variation *CV*_*A*_ and the *I*_*A*_ of the size parameter was calculated after Garcia‐Gonzalez et al. (2012) as simplified by Houle (1992):

$${\mathrm{CV}}_{A}=\frac{\sqrt{V_{A}}}{\text{mean}\;\left(\text{trait}\right)}\;I_{A}={\left(\frac{{\mathrm{CV}}_{A}}{100}\right)}^{2}$$

## 3 RESULTS

### 3.1 16S rRNA sequencing

Of the 17 16s rRNA haplotypes known from New Zealand, only two haplotypes can be found in Europe: haplotypes t and z (Städler et al., 2005). All our Portuguese populations had haplotype z, whereas the remaining Spanish and German populations possessed haplotype t.

### 3.2 Morphological comparison of parental and F1 generations

**FIGURE 3:**
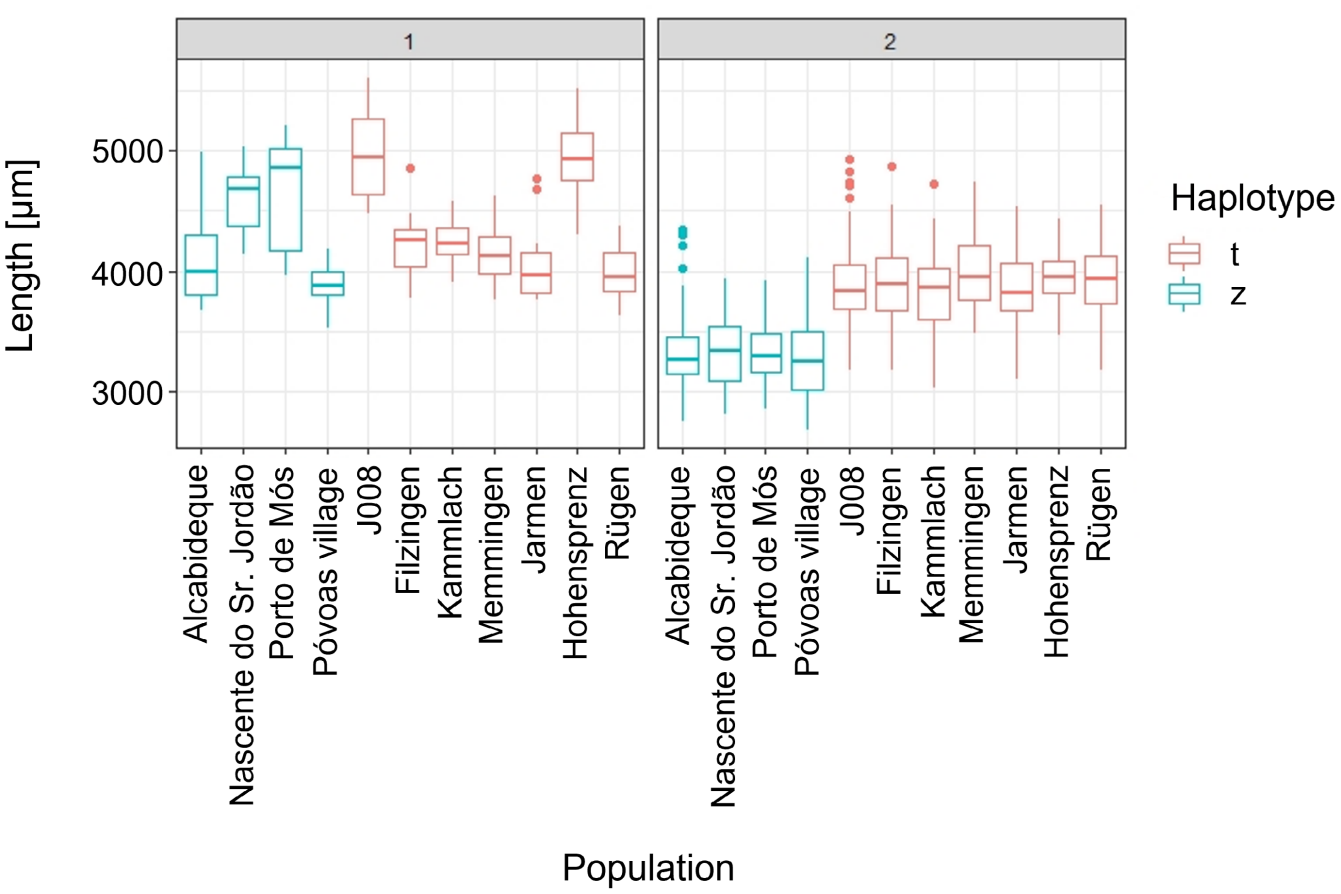
Length across parental (1) and first daughter (2) generations.

The length of the snails of the parental generation distributed across the three climate cabinets, which grew up to finite size under natural and not controlled laboratory conditions, differed across populations but not across haplotypes (Figure 3). The generalized mixed model of length with the lowest AIC was the model with temperature and population as fixed effects. We clearly saw a significant difference in length depending on both population and temperature in the climate cabinets (Table S5). The similar results for centroid size can be seen in Figure S1 and in Table S2. In order to balance the size distribution across temperature treatments, the six smallest mothers were subsequently removed from the analyses regarding the size of the offspring generation as already stated above.

Regarding shape, populations and haplotypes differed significantly along PC 1 (Kruskal–Wallis chi‐squared = 71.73, *df* = 10, *p* < .01; chi‐squared = 51.19, *df* = 1, *p* < .01). PC 2 discriminated only populations (Kruskal–Wallis chi‐squared = 43.92, *df* = 10, *p* < .01) but not haplotypes. Along PC 3, again populations (Kruskal–Wallis chi‐squared = 67.946, *df* = 10, *p* < .01) and haplotypes (Kruskal–Wallis chi‐squared = 49.846, *df* = 1, *p* < .01) were significantly different. For all three PCs, the GLMs with the best AIC were those with population as a fixed effect (Tables S6–S8). The GLMs with the fixed factors population and haplotype had the same AIC, but this was less parsimonious and population and haplotype obviously redundant.

**FIGURE 4:**
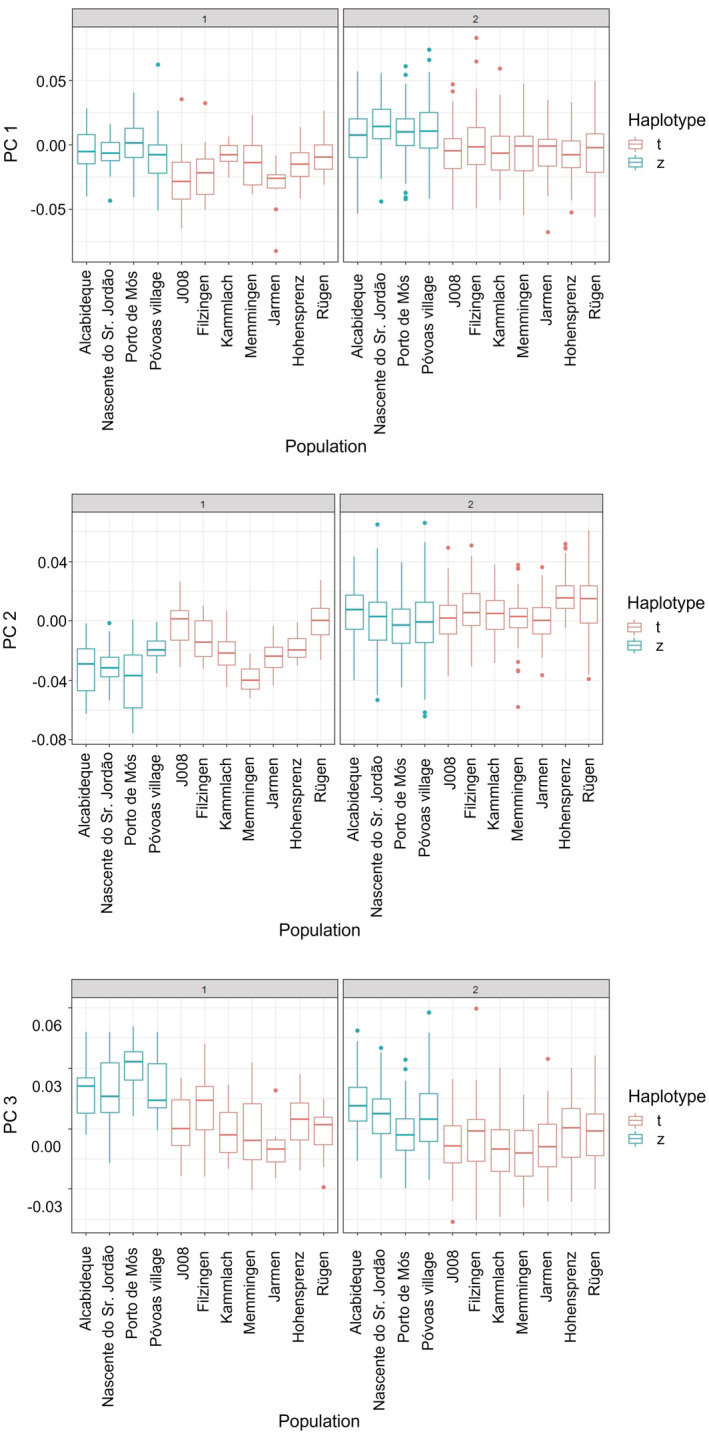
PCs 1 to 3 across parental (1) and first daughter (2) generations.

Concerning the shape analyses, the first offspring generation F1 had a sample size of *n* = 1158, concerning the size analyses a sample size of *n* = 1111. Length and centroid size were significantly different compared with the parent generation [Kruskal–Wallis chi‐squared = 227.84, *df* = 1, *p* < .01 (length); Kruskal–Wallis chi‐squared = 217.41, *df* = 1, *p* < .01 (centroid size)]. Also the shape of the F1 generation was significantly different from the parental generation in all three PCs [Kruskal–Wallis chi‐squared = 59.47, *df* = 1, *p* < .01 (PC 1); Kruskal–Wallis chi‐squared = 188.49, *df* = 1, *p* < .01 (PC 2); Kruskal–Wallis chi‐squared = 38.25, *df* = 1, *p* < .01 (PC 3)]. The shape changes along the first three PCs across parental and F1 generation are illustrated by deformation grids in Figure S6. For both, size (Figure 3) and shape (Figure 4), we observed that the F1 generation became more similar in comparison to the parental generation, although the effect was more pronounced in size.

### 3.3 Morphological traits in the offspring generation

The best‐fitting linear mixed models of length for the F1 generation included the fixed factors temperature, haplotype, and the interaction of temperature and haplotype (Table S9). All models contained the mother snail's ID as a random factor. The interaction of temperature and haplotype showed a significant effect. Similar results can be seen for centroid size (Table S3), apart from the fact that temperature itself already had a significant effect on centroid size. The marginal *R* ^*2*^ of the linear mixed model for length was .55 and the one for centroid size was .42. The conditional *R* ^*2*^ for length was .76 and the one for centroid size was .65. The proportions of variances explained by the random factor, the mother snail, were .21 and .23, respectively.

Regarding shape, the best linear mixed model for PC 1 for the F1 generation was the model with the fixed factor haplotype showing a significant effect (Table S10). The marginal *R* ^*2*^ for PC 1 was .12 and the conditional *R* ^*2*^ was .34. For PC 2, the best model included the fixed factor population and the interaction of temperature and haplotype. Temperature, haplotype, the temperature/haplotype interaction, and all populations except for Hohensprenz and Póvoas village revealed significant effects on PC 2 (Table S11). The marginal and the conditional *R* ^*2*^ were .18 and .49, respectively. Also, the best model for PC 3 with the fixed factors temperature and haplotype showed significant differences (Table S12). The marginal *R* ^*2*^ was .21 and the conditional *R* ^*2*^ was .42. The random factor explained 22%, 31%, and 21% for PCs 1–3, respectively.

### 3.4 Reaction norms

**FIGURE 5:**
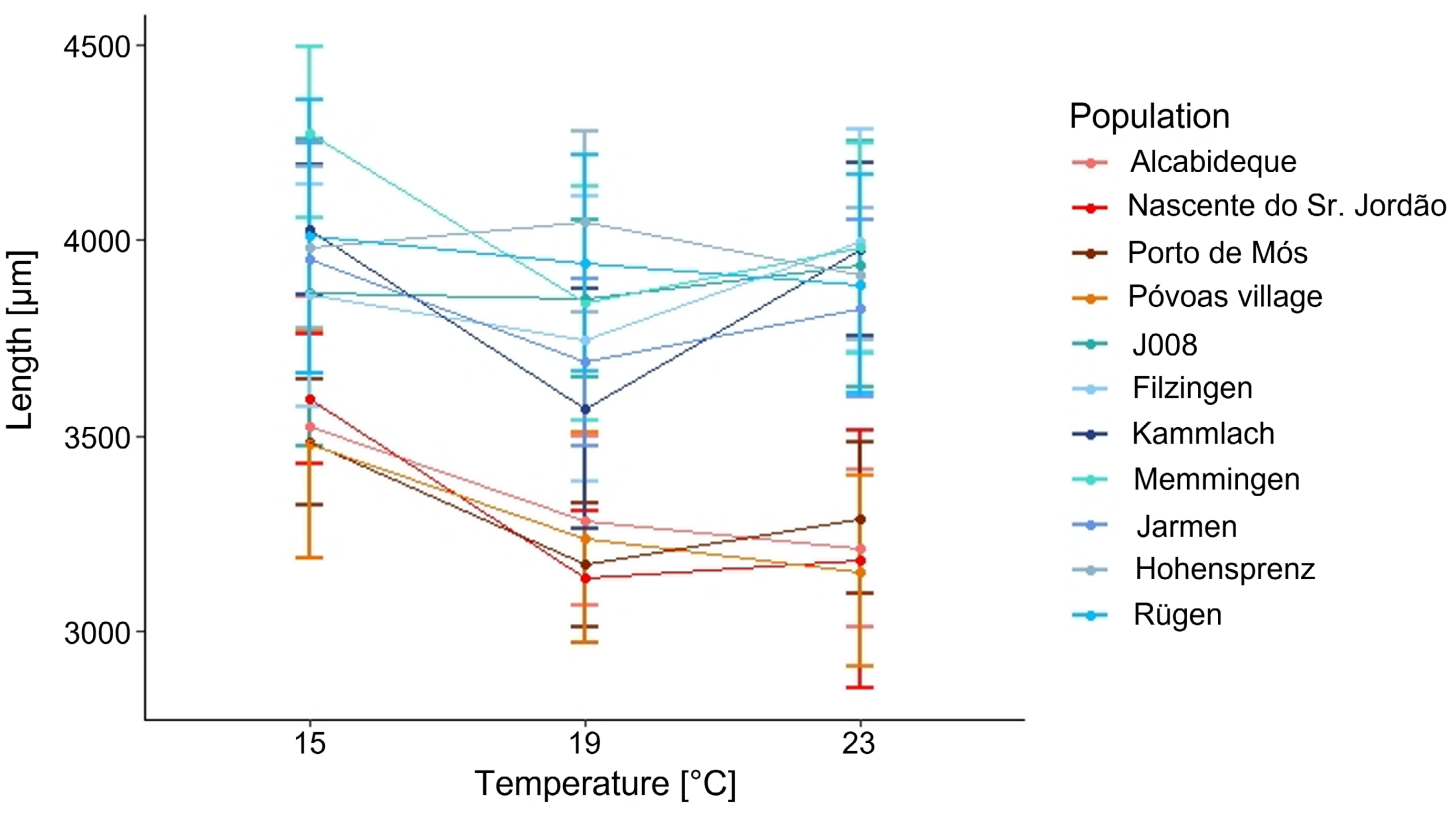
Reaction norms of shell lengths over the three temperatures within generation F1. Populations with haplotype t with bluish line color, populations with haplotype z reddish.

Snails born in the 19 and 23°C climate cabinets were in general smaller than the snails of the coldest 15°C climate cabinet (Figure 5), however, with population and haplotype‐specific reactions. In the comparison of the two haplotypes, snails with haplotype t were in all cases longer and had a larger centroid size than the snails from haplotype z. Regarding the size patterns in within‐population comparison, for the majority of populations (eight out of 11), including all populations with haplotype z, the longest shells developed under the coldest conditions (15°C). For the remaining three populations that deviated from this pattern, the longest shells were found once in the medium temperature (19°C, Hohensprenz), and twice in the warmest temperature conditions (23°C, Filzingen and J008). The pattern where the shortest shells developed was less distinct: in five of the 11 populations, the 19°C snails were the smallest. Pairwise statistical comparisons of slopes revealed a total of 17 significant cases, 15 between the haplotypes, and only one each within either haplotype (Table S13). In summary, shells of both haplotypes tended to be shortest at 19°C and somewhat longer at 15°C than 23°C. However, there was quite some variation among populations.

The pattern for centroid size was similar but not identical. Overall, again the snails of the 19°C climate cabinet had the smallest shells (Figure S2). In contrast to the length, only four (out of 11) populations grew the largest shells at the coldest condition of 15°C: Jarmen and three of the populations with haplotype z. Interestingly, Alcabideque, the last population with haplotype z, showed the largest centroid size in the warmest conditions (23°C). Additional five populations also developed the largest shells at 23°C, viz. Rügen, J008 and all the Southern German snails. Hohensprenz was the only population, where snails were largest at 19°C. For centroid size, 11 pairwise comparisons of the slopes were significant, nine between haplotypes, and one each in either haplotype (Table S4). For centroid size, there was thus a fairly clear picture of shells being smallest at 19°C and of approximately equal size at 15 and 23°C.

**FIGURE 6:**
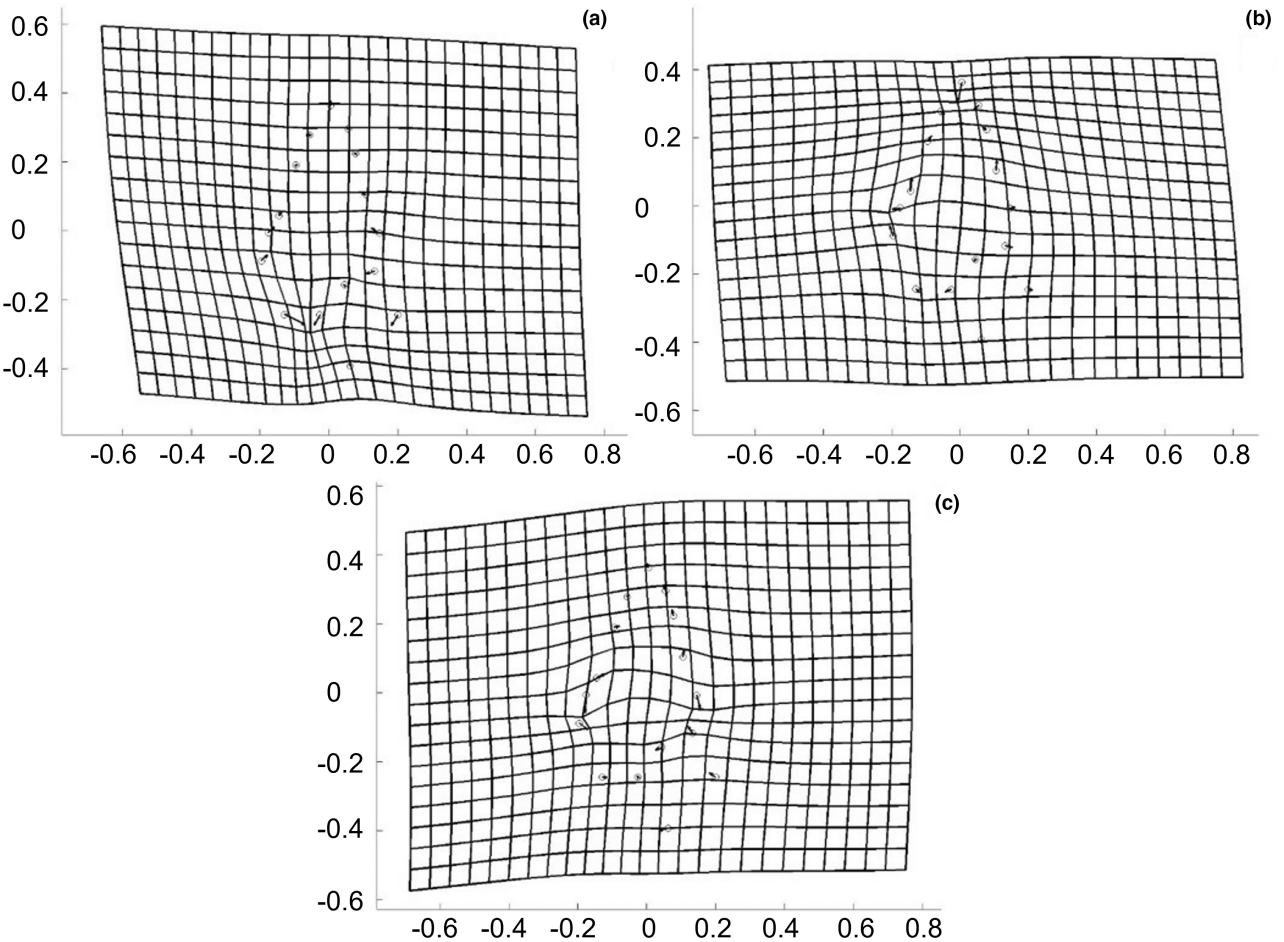
Deformation grids for PC 1 (a), PC 2 (b), and PC 3 (c) of the F1 generation. Grids show deformation from specimens with lowest (circle) to those with highest scores (arrowhead).

**FIGURE 7:**
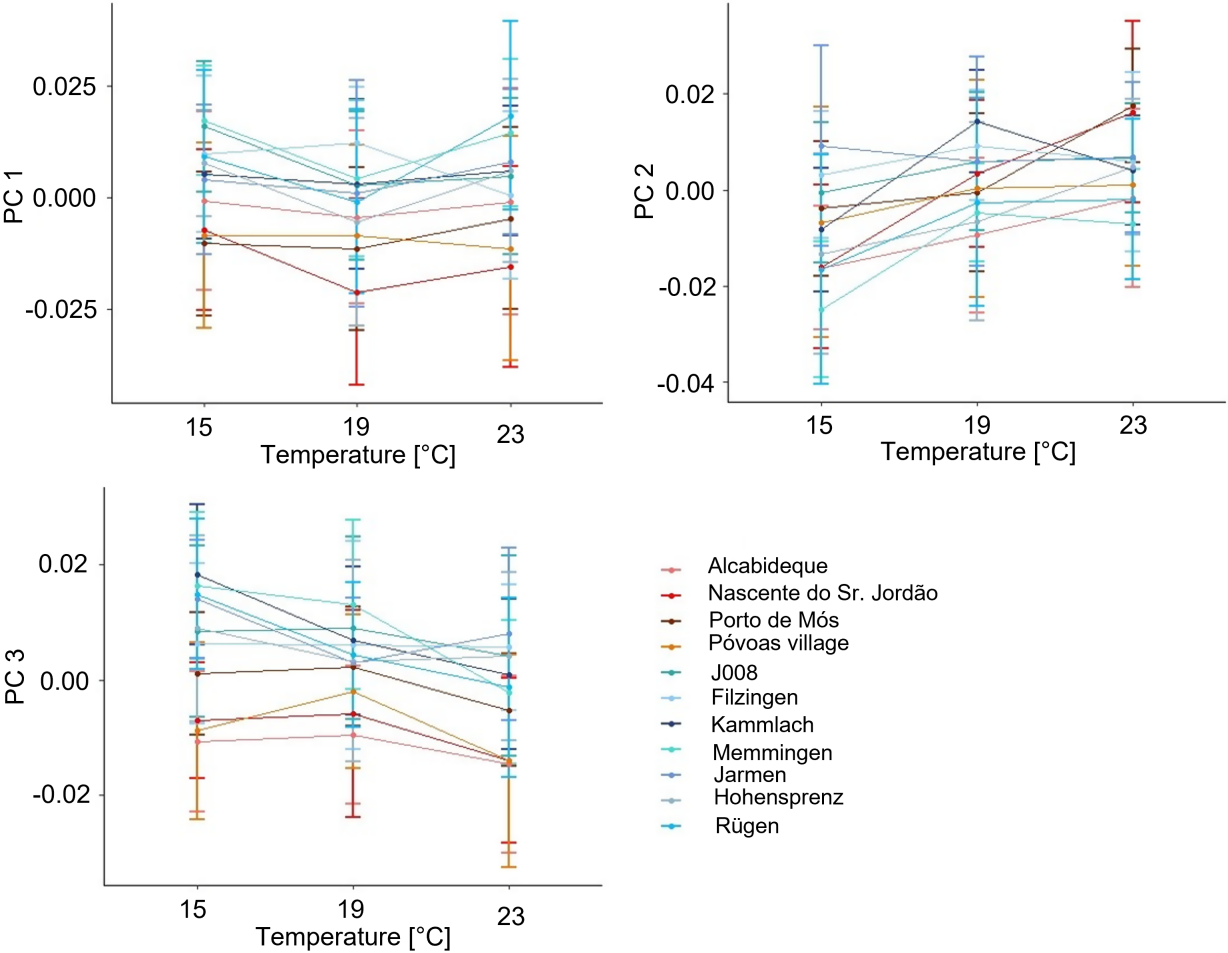
Reaction norms of PC 1, 2, and 3 across the three temperatures within generation F1. Populations with haplotype t with bluish line color, populations with haplotype z reddish.

Reaction norms for shape are summarized in Figure 7 and shape changes along the first three PCs are visualized by the deformation grids in Figure 6. Along PC 1, the lower parts of the last whorl and the aperture became narrower. Along PC 2, the spire became shorter and the body whorl larger. And PC 3 described a change toward a higher body whorl. For PC 1 and PC 3, populations possessing haplotype z had smaller values than snails from populations with haplotype t. For PC 1, the snails kept at 19°C showed the lowest scores, indicating more globular lower parts of the last whorl and apertures in all populations except for Filzingen and Póvoas village. In four populations, the 15°C snails had higher values. Five populations showed the highest values at 23°C. Only the snails from Filzingen had the maximum at 19°C (Figure 7).

Along PC 2, all populations with haplotype z had increasing scores with increasing temperatures. By contrast, all populations with haplotype t except Jarmen had their maximum at either 19 or 23°C and these values tended to be very similar with the exception of Kammlach (Figure 7).

For PC 3 the picture was again more heterogeneous. Six populations with haplotype t had the highest scores at 15°C, and five of them had the minimum at 23°C. J008 had the maximum at 19°C, and the Filzingen snails hardly differed at all across the three temperatures. All four haplotype z populations had their maximum at 19°C, with very similar values at 15°C, though, and the minimum at 23°C (Figure 7).

Shape changes were generally subtle with considerably less differences in slopes compared with size (Tables S14–S16). For PC 1 there were only three significant pairwise differences, two within haplotype t and one between haplotypes. PC 2 was somewhat more informative revealing seven significant comparisons between haplotypes and two between populations with haplotype z. For PC 3 we detected only a single significant case within haplotype t. In general, snails with haplotype z had more squatter shells than those with haplotype t. Snails of haplotype t tended to have a wider aperture and base of the final whorl at 19°C and the spire became shorter and the body whorl larger and in particular higher with increasing temperature. Shape changes in haplotype z were quite similar albeit more pronounced compared to those observed in haplotype t with the base of the final whorl and aperture becoming narrower, the spire shorter and the body whorl larger and higher.

### 3.5 Heritability

Heritabilities, coefficients of genetic variance, and *I*_*A*_ for the different temperatures of the F1 generation are summarized in Tables S17–S19.

Heritabilities for both size parameters, length and centroid size, were fairly high ranging from 0.49 to 0.80. Thus, the overall variation observed was largely due to genetic differentiation. Values were smaller at 15°C than at 19 and 23°C. By contrast, heritability estimates for shape were smaller than for size and highest at 15°C.

The *CV*_*A*_ values were slightly larger for shell length than for centroid size but, in general, very low ranging from 0.39% to 1.13%. Together with the corresponding low *I*_*A*_ values, this indicates a very low potential to respond to selection, i.e., low evolvability, in particular at the coldest temperature condition of 15°C.

Assuming that the relatively high heritability estimates were in large parts driven by differences across the haplotypes, we also calculated the heritabilities for each of the two haplotypes separately. As expected, the resulting haplotype‐specific values were generally considerably lower (Tables S20–S25). For size, heritabilities now ranged from 0.29 to 0.59 in haplotype t and 0.24–0.37 in z. In the former, they were lowest at 23°C and in the latter almost invariant across temperatures. *CV*_*A*_ values all dropped below 1%. For shape, the picture was similar except that heritabilities were lowest at 19°C.

### 3.6 Life history

The reproductive rates of the snails in the parental generation were only significantly different across haplotypes (Kruskal–Wallis chi‐squared = 45.311, *df* = 24, *p* < .01). Temperature and temperature differences showed a high correlation. The best‐fitting generalized linear model was the one including temperature in the climate cabinets, haplotype, and their interaction as fixed factors. All three, temperature, haplotype, and the interaction of both had a significant influence on the reproductive rate of the mother snails (Table S31). Mother snails with haplotype z had a faster reproductive rate than individuals with haplotype t. We saw this pattern across all temperatures. Temperature had a significant influence on mother snails with haplotype t (Kruskal–Wallis chi‐squared = 11.929, *df* = 2, *p* < .01) but not on mother snails with haplotype z. Within haplotype t, only the snails from the 15°C and the 23°C climate cabinets showed a significant difference (Figure S7). The mean reproductive rate with haplotype t was 566.28 days and with haplotype z 467.71 days, which means that snails with haplotype z finished their reproduction around 99 days earlier than snails with haplotype t.

## 4 DISCUSSION

In our common garden experiment, we investigated the influence of temperature on shell morphology and reproduction across 11 populations of the clonal, invasive NZMS, *P. antipodarum*. Our particular interest lay in disentangling genetic and phenotypically plastic responses with the ultimate goal to assess the potential of the European populations for genetic adaptation after having experienced a drastic bottleneck at the time of establishment 180–360 generations ago (Ponder, 1988; Verhaegen et al., 2021). The analyses were conducted against the background of the rising temperatures Europe is facing under current climate change scenarios (Masson‐Delmotte et al., 2021).

### 4.1 Morphological traits

The parental populations were morphologically very variable. This variation in length and shape was mainly population‐specific, while differences explained by the haplotype were visible only in shape. As already reported by Kistner and Dybdahl (2013) who investigated three North American populations of the NZMS, daughter snails raised in the common garden were smaller than their parents and became more similar to each other, possibly as a response to the lack of water flow (Verhaegen, McElroy, et al., 2018). However, among our European populations, marked differences remained between the haplotypes, particularly in terms of size, whereas more population‐specific differences remained among the US populations. Also in the sea snail *Monetaria annulus* the size difference between wild populations vanished among offspring raised in a common garden (Irie & Morimoto, 2008). The general findings of shells becoming more similar to each other under identical conditions is in accordance with our expectations assuming limited genetic differentiation among European NZMS. Hence, the differences observed among the parental samples were largely site‐specific responses due to phenotypic plasticity.

Snails of the F1 generation not only differed across haplotypes but also across temperatures. Temperature and haplotype explained between 42% and 55% of the variation in shell size. In general, size at 15°C was larger than at 19 and 23°C. This thermal plasticity is in accordance with the temperature‐size‐rule, which is based on observations across many ectotherm taxa extending their growth period at lower temperatures, thus delaying reproduction and maturing at larger body size (Angilletta et al., 2004; Atkinson, 1994, 1995; Atkinson et al., 2003). The adaptive significance of our findings probably lies in the fact that larger snails sire more offspring (Verhaegen, McElroy, et al., 2018), thus compensating for the lower growth rate and delayed maturation. However, our observations may well be due to a multivariate interplay of reproductive traits (Angilletta et al., 2004) whose investigation requires further thorough experimentation. Differences in slopes were mainly detected between haplotypes, with 15 of the significant pairwise comparisons occurring between haplotypes and just one each within haplotypes. This indicates genetic differentiation between haplotypes t and z regarding the plastic thermal reaction of size but only limited population‐specific variation (Pigliucci, 2005). Thus, our results with respect to the temperature dependence of size were similar to the findings of Dybdahl and Kane (2005), who compared the reaction norms of three North American populations of the NZMS in a common garden at three temperatures. The comparability of both studies is limited, though, as Dybdahl and Kane (2005) set a temporal limit to their experiment whereas our criterion, attaining final size, was a morphological one.

In terms of shape, snails of haplotype z were generally squatter than those carrying t as already observed by Verhaegen, McElroy, et al. (2018). Daughter snails raised in the common garden had higher scores on PCs 1 and 2 and lower ones along PC 3 than their parents, translating into more slender shells with a relatively smaller body whorl (Figure S6). They were again more similar to each other than the snails from the parental generation. The factors investigated explained considerably less variation in shape than in size. Haplotype accounted for only 12% along PC 1, haplotype, population and temperature explained 18% of the variation of PC 2, and 21% of the variation along PC 3 were due to haplotype and temperature. This indicates that most of the variation in shape explained by the fixed factors was due to genetic differentiation. The reaction norms also showed much less variation with three, nine, and one significant pairwise comparisons along PCs 1–3, respectively. Thus, plastic responses were more restricted for shape than for size. In both haplotypes, the body whorl housing the distal genitalia, where embryos are brooded, became larger relative to the spire with rising temperature, in snails with haplotype z more pronounced than in those with haplotype t. This result is thus not in accordance with our expectations assuming a thermoregulatory function of the shell shape (Albarrán‐Mélzer et al., 2020; Wong & Lim, 2017). Rather, this response may compensate for the decrease in size, which followed the temperature‐size rule (Angilletta et al., 2004; Atkinson, 1994, 1995; Atkinson et al., 2003) to provide more space for the developing embryos. The subtlety of the response is possibly a consequence of the limited genetic variation curbing the phenotypically plastic reaction as predicted by the frozen‐phenotype variation model (Jokela et al., 1997), although we do not know the variation of the phenotypic plastic reaction of shape to temperature across a larger number of clones.

Investigations of plastic responses of shell morphology to temperature in freshwater gastropods are generally still scarce despite global warming (see the review of Whelan, 2021). The hydrobiid *Pyrgophorus coronatus* native to lakes in southern Mexico developed more globular shells at lower temperatures while the thiarid *Tarebia granifera*, invasive to the same habitats, was morphologically hardly affected. The response in the former was related to temperature regulation (Albarrán‐Mélzer et al., 2020). By contrast, *Physa virgata* (a synonym of *Physella acuta*, Physidae) from North America, developed more slender shells at lower temperatures (Britton & McMahon, 2004). However, the authors did not provide a functional explanation. Larger and thinner shells with wider apertures were observed at higher temperatures in the ampullariid *Pomacea canaliculata*. This was attributed to increased growth rates at higher temperatures (Tamburi et al., 2018). Although there are only these few examples, it is already obvious that different species may respond differently to increasing temperatures. This is not surprising as the responses probably evolved under different combinations of proximate and ultimate causes. More comprehensive insights into the variation of plasticity certainly require the experimental consideration of a combination of factors.

### 4.2 Maternal ID

Maternal effects, the causal influence of the maternal genotype (other than through direct inheritance, *specification ours*) or phenotype on the offspring phenotype (Wolf & Wade, 2009), have been reported in North American populations of the NZMS by Dybdahl and Kane (2005) for size and age at first reproduction. For shell shape, maternal effects were not detected by Kistner and Dybdahl (2013), but Smithson et al. (2020) did report evidence for stable shape in different environments across one generation. These studies used different approaches to quantify shape, though. We included “maternal ID” as a random factor in our models. This random factor accounted for roughly 20% of the variance, in PC 2 for shape even for 31%. However, this variable comprises more than potential maternally transmitted factors. As the offspring stayed in the same jar with their mother, we are not able to distinguish the impact of the mother snail from that of the environment in the corresponding jar. Although we aimed at keeping the conditions identical across the entire experimental setup, it was not possible to standardize all factors. As mother snails reproduced at different rates snails experienced different population densities, which are known to affect life history traits in the NZMS (Cope & Winterbourn, 2004; Neiman et al., 2013; Zachar & Neiman, 2013). Also, it was impossible to adjust the amount of food to these population compositions differing across jars (see e.g., Neiman et al., 2013). A third inconsistency we were aware of but could not avoid was the differential amount and composition of epiphytic algae the parental snails imported on their shells which possibly interfered with our feeding.

While we were able to account for a potential maternal effect in our mixed models, we could not entirely exclude an effect on the reaction norms. Consequently, a maternal effect may play a role in the differences that were observed. Still, three lines of evidence suggest that true maternal effects did probably not play a major role in shaping the morphologies. (1) Already the F1 generation differed considerably from the parental one, in particular in size. (2) F1 snails from different populations became similar to each other despite the different conditions their mothers experienced. And (3) the limited results for the snails of the F2 generation from Portuguese lineages did not differ substantially from those for the F1 snails (Appendix S6). Therefore, if the founding mothers did cause a non‐Mendelian effect, it must have been stable across more than one generation, e.g., through heritable epigenetic modifications (Heard & Martienssen, 2014; Ho & Burggren, 2010; Youngson & Whitelaw, 2008), and probably smaller than the environmental effects of the common garden. The detection of an eventual effect would require experimentation across further generations, which is logistically very challenging.

### 4.3 Heritability

The heritability estimates of 0.49–0.80 in the total population for size were comparatively high (Dybdahl & Kane, 2005; for a more general overview see Fischer et al., 2021) suggesting a high degree of genetic determination. Based on the comparison of parental snails, which grew under natural conditions, and the F1 generation raised in the climate cabinets (Figure 3), we would have expected much lower values. This, however, indicates that the contribution of the temperature differences to the total variance among F1 snails was comparatively low. The high morphological variation observed among European natural populations is certainly due to the effects of a larger combination of environmental factors (Verhaegen, McElroy, et al., 2018). In our experiment, temperature was the only specifically manipulated factor. Haplotype‐specific heritability estimates were considerably lower approaching more commonly reported values (Fischer et al., 2021).

Heritabilities for shape were considerably lower than for size, which was again somewhat unexpected as previous studies based on field data suggested that genetic variation underlying shape was more important than for size (Verhaegen, McElroy, et al., 2018; Verhaegen, Neiman, et al., 2018). This seeming discrepancy may be due to the nature of estimating heritability. Our models indicated that only a small part of the variation was explained by the factors included. 21%–31% of the variance were attributed to “maternal ID” (see below), and more than 50% remained unexplained, yet, were component of the total phenotypic variation, hence, possibly responsible for the low heritability values (Garcia‐Gonzalez et al., 2012; Hansen et al., 2011; Houle, 1992). This exemplifies the general problems of comparability of heritability estimates (Hansen et al., 2011; Houle, 1992).

Heritabilities were sensitive to different temperatures. This held in particular for snails with haplotype t. Thus, shell morphology depends on the interplay of both genetic factors and environmental factors including temperature. Their relative contributions to the variation in morphology differ depending on the environmental conditions a population experiences and its genetic diversity.

As already addressed above, heritability is not an appropriate measure to compare evolvabilities. Such inferences are better based on measures of genetic variation standardized by trait means rather than the total phenotypic variance such as the coefficient of genetic variation *CV*_*A*_ and its square *I*_*A*_ (Garcia‐Gonzalez et al., 2012; Hansen et al., 2011; Houle, 1992). As invasive lineages of the NZMS are parthenogenetic, we here replaced the additive genetic variance with clonal repeatability. Values for these measures were very low indicating that the European populations are genetically deprived. Not much variation has accumulated since the species arrival in Europe suggesting that there is limited potential to respond to selection.

### 4.4 Life history

Reproductive rate—the time until the number of offspring required for the experiment was sired—was mainly dependent on the haplotype and the temperature of the climate cabinets. Mother snails with haplotype z reproduced faster than mother snails with haplotype t. The reproductive rate of snails with haplotype t was more affected by the temperature of the climate cabinets than snails with haplotype z (Figure S7). Therefore, snails with haplotype t seem to be less buffered against changing temperatures than snails with haplotype z. Temperature difference between temperature measured in the habitat at collecting and the climate cabinets did not affect the reproductive rate suggesting that the time for acclimation to the lab conditions prior to the experiment was sufficient to counteract eventual maternal effects. Other studies reported growth rates to increase with temperature up to around 24°C and then drop again, i.e., they follow an optimum curve (Bennett et al., 2015; Dybdahl & Kane, 2005), which is largely in accordance with our findings for the snails with haplotype t. The reproductive rate obviously has a variable genetic basis but may be tuned through phenotypic plasticity. The extent of the plasticity itself seems to depend on the genetic background as well. Several previous studies showed the importance of phenotypic plasticity in life history traits in the NZMS (e.g., Bennett et al., 2015; Dybdahl & Kane, 2005; Kistner & Dybdahl, 2013; McKenzie et al., 2013; Negovetic & Jokela, 2001). Field observations showed that the North German populations from Hohensprenz, Jarmen, and Rügen, all with haplotype t and also included here, differed in reproductive characteristics, which was linked to environmental influences (Verhaegen et al., 2021). On the other hand, limitations to plasticity in clonal organisms would be expected according to the frozen‐phenotypic‐variation model (Jokela et al., 1997), a modification of the frozen‐niche‐variation model (Vrijenhoek, 1984), and flat reaction norms have also been reported for some life history traits in the NZMS (Jokela et al., 1997).

## 5 CONCLUSIONS

Our experiment confirmed the generally high capacity of the NZMS to adjust its shell morphology through phenotypic plasticity with major differences between the two clonal lineages present in Europe. In particular size, and to a more limited extent also shape, were sensitive to temperature, the focal factor in our analyses. The interaction of temperature and haplotype explained about 50% of the total variance in size and we observed more population differentiation than shape. Across the three temperatures, size followed the expectations of the temperature‐size rule (Angilletta et al., 2004). Changes in shape may have compensated for changes in size affecting space for brooding embryos in the distal genitalia. The relatively high values for our heritability estimates can probably not be compared in absolute terms considering the very low *CV*_*A*_ values. However, they showed some sensitivity to temperature, in haplotype t more so than in z. The low *CV*_*A*_ values indicate that genetic variation among European populations is still restricted with low potential to react to selection despite some differentiation detected in the reaction norms. A large amount of the genetic variation was due to differences between the clonal lineages. Thus, 180–360 generations presence in Europe was apparently not sufficient to accumulate significant genetic variation relevant for morphological adaptation. On the other hand, genetic variation increasing the morphological diversity beyond the variation we have observed so far was apparently not necessary to conquer Europe.

In Europe, the haplotypes differ in salinity preferences with z occurring in brackish waters and t dominating in freshwaters. Only rarely and only in coastal vicinity are both encountered sympatrically (Butkus et al., 2020; Verhaegen, McElroy, et al., 2018). Therefore, it was surprising that in Portugal haplotype z has established far inland in freshwater. In our common garden, the reproductive rate of mother snails possessing haplotype z was faster and suggested a competitive advantage over haplotype t. This, however, would need experimental confirmation. The artificial salinity of the common garden experiment of 0.5‰ was in the upper range of salinities measured in natural habitats, however, still represents freshwater. As salinity varies constantly in natural habitats and the NZMS has a high salinity tolerance including brackish water (Hoy et al., 2012; Verhaegen et al., 2021), an influence of the salinity in our experiment can probably be excluded.

The relevance of laboratory assays investigating development at constant temperatures for natural populations has repeatedly been questioned. However, several studies confirmed that such laboratory data can at least to some extent be extrapolated (e.g., Fischer et al., 2011; von Schmalensee et al., 2021 and literature therein). In any case, we have shown that temperature affects shell morphology. It is also clear, though, that variation in temperature is only one factor and probably not the dominating one contributing to the morphological variation observed in natural populations. The importance of temperature relative to other environmental factors has to be quantified in further studies. The fact that temperature is not the sole factor influencing shell morphology and reproduction also suggests that their interaction will not be a factor limiting the population persistence of the NZMS under a warming climate in Europe. Physiological processes directly influencing reproduction will probably respond more sensitively to higher temperatures (Dybdahl & Kane, 2005; Quinn et al., 1994; Winterbourn, 1970).

## AUTHOR CONTRIBUTIONS

**Lisa Männer:** Data curation (lead); formal analysis (lead); investigation (equal); methodology (lead); project administration (lead); visualization (equal); writing – original draft (lead); writing – review and editing (equal). **Carolin Mundinger:** Formal analysis (equal); writing – original draft (supporting); writing – review and editing (equal). **Martin Haase:** Conceptualization (lead); funding acquisition (lead); investigation (supporting); methodology (supporting); supervision (lead); writing – original draft (supporting); writing – review and editing (equal).

## FUNDING INFORMATION

All three authors were involved in the Research Training Group 2010 RESPONSE funded by the Deutsche Forschungsgemeinschaft.

Open Access funding enabled and organized by Projekt DEAL.

## CONFLICT OF INTEREST

We declare we have no competing interests.

## Supporting information


Data S1

## Data Availability

Data used in this study are available at the Dryad Digital Repository (https://doi.org/10.5061/dryad.kh189328w).
