# Supplementary material for: Stay in shape: Assessing the adaptive potential of shell morphology and its sensitivity to temperature in the invasive New Zealand mud snail Potamopyrgus antipodarum through phenotypic plasticity and natural selection in Europe
Source: Ecol Evol. 2022 Oct 1;12(10):e9314. doi: 10.1002/ece3.9314 (PMC9526036; doi:10.1002/ece3.9314)
Supplement: Supplementary file 1 — Data S1 [file ECE3-12-e9314-s001.docx]

**Appendix**

**Appendix 1: Variance of shape explained by PCAs**

**Supplemental Table S1.** Variance explained by first 3 principal components (PC) based on uncorrected data. P, parental generation, F, daughter generation.

|  | **PC 1** | **PC 2** | **PC 3** | **Sum** |
| --- | --- | --- | --- | --- |
| **P + F1 + F2** | 0.298 | 0.247 | 0.123 | 0.668 |
| **P + F1** | 0.225 | 0.197 | 0.139 | 0.561 |
| **P** | 0.241 | 0.169 | 0.128 | 0.538 |
| **F1** | 0.237 | 0.177 | 0.136 | 0.55 |
| **F2** | 0.319 | 0.203 | 0.109 | 0.631 |

**Appendix 2: Results for centroid size**


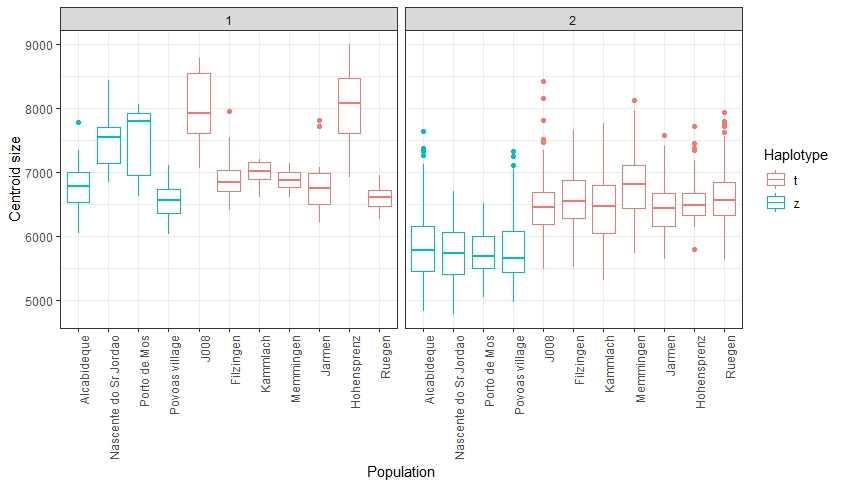


**Supplemental Figure S1.** Centroid size across all the three generations depending on haplotype and population [(Kruskal-Wallis chi-squared 92.29, df = 10, p < 0.001 (population); Kruskal-Wallis chi-squared 0.25, df = 1, p = 0.62 (haplotype)].

**Supplemental Table S2.** Generalized linear model of centroid size including the fixed factors temperature and population in the parental generation. Significance codes: 0 ‘***’ 0.001 ‘**’ 0.01 ‘*’ 0.05 ‘.’ 0.1 ‘ ’ 1.

|  | **Estimate** | **Standard error** | **t-value** | **p-value** |
| --- | --- | --- | --- | --- |
| **Intercept** | 6935.206 | 106.019 | 65.415 | < 2e-16 *** |
| **19°C** | -191.902 | 81.462 | -2.356 | 0.01981 * |
| **23°C** | -230.054 | 76.925 | -2.991 | 0.00327 ** |
| **Filzingen** | 130.950 | 142.496 | 0.919 | 0.35962 |
| **Hohensprenz** | 1207.240 | 145.812 | 8.279 | 6.96e-14 *** |
| **J008** | 1199.862 | 139.984 | 8.571 | 1.29e-14 *** |
| **Jarmen** | -2.007 | 142.734 | -0.014 | 0.98880 |
| **Kammlach** | 179.281 | 152.434 | 1.176 | 0.24145 |
| **Memmingen** | 92.245 | 139.985 | 0.659 | 0.51095 |
| **Nascente do Sr. Jordão** | 673.497 | 142.734 | 4.719 | 5.48e-06 *** |
| **Porto de Mós** | 647.051 | 145.398 | 4.450 | 1.68e-05 *** |
| **Póvoas village** | -269.398 | 152.409 | -1.768 | 0.07920 . |
| **Rügen** | -193.266 | 148.301 | -1.303 | 0.19454 |

**Supplemental Table S3.** Linear mixed model of centroid size including the fixed factors temperature and haplotype and the random factor of the mother snail´s ID in the F1 generation. Significance codes: 0 ‘***’ 0.001 ‘**’ 0.01 ‘*’ 0.05 ‘.’ 0.1 ‘ ’ 1.

|  | **Estimate** | **Standard error** | **df** | **t-value** | **p-value** |
| --- | --- | --- | --- | --- | --- |
| **Intercept** | 6101.19 | 209.75 | 160.61 | 29.089 | < 2e-16 *** |
| **Temperature** | 22.73 | 10.60 | 157.73 | 2.145 | 0.0335 * |
| **Haplotype z** | -18.55 | 316.00 | 156.59 | -0.059 | 0.9533 |
| **Temperature x Haplotype z** | -39.09 | 16.22 | 154.93 | -2.410 | 0.0171 * |


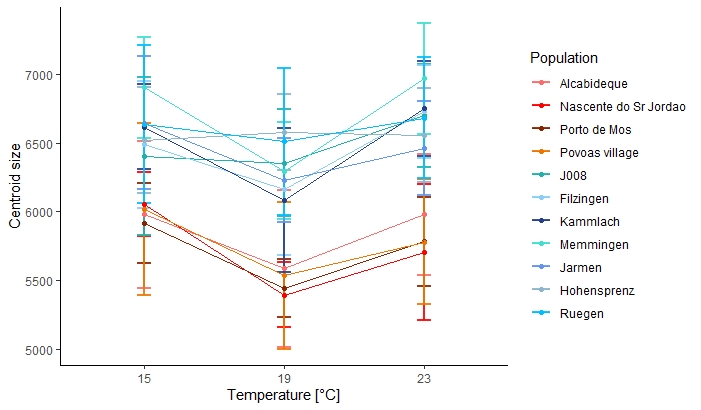


**Supplemental Figure S2.** Reaction norm of the mean centroid size over the three temperatures within generation F1. Populations are marked with different colours. Populations with haplotype t have a bluish line colour, populations with haplotype z a reddish line colour.

**Supplemental Table S4.** Comparison of reaction norm slopes of centroid size across populations of the F1 generation. Significant differences (p < 0.05) are written in bold.


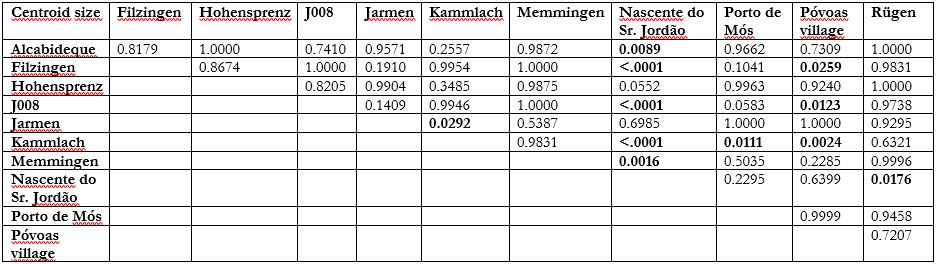


**Appendix 3: GLMs of the morphological comparison of parental and F1 generations**

**Supplemental Table S5**. Generalized linear model of length including the fixed factors temperature and population in the parental generation. Significance codes: 0 ‘***’ 0.001 ‘**’ 0.01 ‘*’ 0.05 ‘.’ 0.1 ‘ ’ 1.

|  | **Estimate** | **Standard error** | **t-value** | **p-value** |
| --- | --- | --- | --- | --- |
| **Intercept** | 4193.47 | 79.12 | 53.001 | < 2e-16 *** |
| **Temperature 19°C** | -174.21 | 60.79 | -2.866 | 0.00477 ** |
| **Temperature 23°C** | -130.46 | 57.41 | -2.272 | 0.02451 * |
| **Filzingen** | 127.10 | 106.34 | 1.195 | 0.23395 |
| **Hohensprenz** | 819.70 | 108.82 | 7.533 | 4.66e-12 *** |
| **J008** | 889.59 | 104.47 | 8.515 | 1.79e-14 *** |
| **Jarmen** | -50.44 | 106.52 | -0.474 | 0.63652 |
| **Kammlach** | 143.48 | 113.76 | 1.261 | 0.20921 |
| **Memmingen** | 58.39 | 104.47 | 0.559 | 0.57706 |
| **Nascente do Sr. Jordão** | 486.66 | 106.52 | 4.569 | 1.03e-05 *** |
| **Porto de Mós** | 535.55 | 108.51 | 4.936 | 2.14e-06 *** |
| **Póvoas village** | -238.53 | 113.74 | -2.097 | 0.03770 * |
| **Rügen** | -102.19 | 110.68 | -0.923 | 0.35736 |

**Supplemental Table S6**. Generalized linear model of PC 1 for shape including the fixed factor population in the parental generation. Significance codes: 0 ‘***’ 0.001 ‘**’ 0.01 ‘*’ 0.05 ‘.’ 0.1 ‘ ’ 1.

|  | **Estimate** | **Standard error** | **t-value** | **p-value** |
| --- | --- | --- | --- | --- |
| **Intercept** | 0.0140459 | 0.0043153 | 3.255 | 0.001393 ** |
| **Filzingen** | -0.0373179 | 0.0062906 | -5.932 | 1.88e-08 *** |
| **Hohensprenz** | -0.0223248 | 0.0065241 | -3.422 | 0.000795 *** |
| **J008** | -0.0160821 | 0.0062906 | -2.557 | 0.011532 * |
| **Jarmen** | -0.0128257 | 0.0064006 | -2.004 | 0.046831 * |
| **Kammlach** | -0.0375478 | 0.0068231 | -5.503 | 1.51e-07 *** |
| **Memmingen** | -0.0212181 | 0.0062906 | -3.373 | 0.000939 *** |
| **Nascente do Sr. Jordão** | 0.0004233 | 0.0064006 | 0.066 | 0.947355 |
| **Porto de Mós** | 0.0138798 | 0.0064006 | 2.169 | 0.031644 * |
| **Póvoas village** | -0.0060304 | 0.0062906 | -0.959 | 0.339231 |
| **Rügen** | -0.0233737 | 0.0066637 | -3.508 | 0.000592 *** |

**Supplemental Table S7**. Generalized linear model of PC 2 for shape including the fixed factor population in the parental generation. Significance codes: 0 ‘***’ 0.001 ‘**’ 0.01 ‘*’ 0.05 ‘.’ 0.1 ‘ ’ 1.

|  | **Estimate** | **Standard error** | **t-value** | **p-value** |
| --- | --- | --- | --- | --- |
| **Intercept** | -0.0058858 | 0.0040231 | -1.463 | 0.145492 |
| **Filzingen** | 0.0202313 | 0.0058646 | 3.450 | 0.000723 *** |
| **Hohensprenz** | 0.0139366 | 0.0060823 | 2.291 | 0.023292 * |
| **J008** | 0.0034443 | 0.0058646 | 0.587 | 0.557858 |
| **Jarmen** | -0.0117383 | 0.0059672 | -1.967 | 0.050952 . |
| **Kammlach** | -0.0057496 | 0.0063610 | -0.904 | 0.367461 |
| **Memmingen** | 0.0102367 | 0.0058646 | 1.746 | 0.082878 . |
| **Nascente do Sr. Jordão** | -0.0003347 | 0.0059672 | -0.056 | 0.955348 |
| **Porto de Mós** | -0.0010598 | 0.0059672 | -0.178 | 0.859264 |
| **Póvoas village** | 0.0116155 | 0.0058646 | 1.981 | 0.049405 * |
| **Rügen** | 0.0245915 | 0.0062125 | 3.958 | 0.000115 *** |

**Supplemental Table S8**. Generalized linear model of PC 3 for shape including the fixed factor population in the parental generation. Significance codes: 0 ‘***’ 0.001 ‘**’ 0.01 ‘*’ 0.05 ‘.’ 0.1 ‘ ’ 1.

|  | **Estimate** | **Standard error** | **t-value** | **p-value** |
| --- | --- | --- | --- | --- |
| **Intercept** | 0.008601 | 0.003218 | 2.672 | 0.008335 ** |
| **Filzingen** | -0.015930 | 0.004692 | -3.395 | 0.779960 |
| **Hohensprenz** | -0.001362 | 0.004866 | -0.280 | 0.779960 |
| **J008** | -0.020800 | 0.004692 | -4.433 | 1.75e-05 *** |
| **Jarmen** | -0.015057 | 0.004774 | -3.154 | 0.001934 ** |
| **Kammlach** | -0.013851 | 0.005089 | -2.722 | 0.007237 ** |
| **Memmingen** | -0.015991 | 0.004692 | -3.408 | 0.000833 *** |
| **Nascente do Sr. Jordão** | 0.002309 | 0.004774 | 0.484 | 0.629292 |
| **Porto de Mós** | 0.007071 | 0.004774 | 1.481 | 0.140583 |
| **Póvoas village** | 0.002207 | 0.004692 | 0.470 | 0.638669 |
| **Rügen** | -0.026137 | 0.004970 | -5.259 | 4.74e-07 *** |

**Appendix 4: LMMs of the morphological traits in the offspring generation**

**Supplemental Table S9.** Linear mixed model for shell length including the fixed factors temperature, haplotype and the interaction of temperature and haplotype as well as the random factor of the mother snail´s ID in the F1 generation. Significance codes: 0 ‘***’ 0.001 ‘**’ 0.01 ‘*’ 0.05 ‘.’ 0.1 ‘ ’ 1.

|  | **Estimate** | **Standard error** | **df** | **t-value** | **p-value** |
| --- | --- | --- | --- | --- | --- |
| **Intercept** | 3905.90 | 132.16 | 155.30 | 29.55 | < 2e-16 *** |
| **Temperature** | 0.34 | 6.68 | 152.99 | 0.05 | 0.96 |
| **Haplotype z** | 127.26 | 199.36 | 152.36 | 0.64 | 0.52 |
| **Temperature x Haplotype z** | -38.18 | 10.24 | 151.06 | -3.73 | 0.00027 *** |

**Supplemental Table S10.** Linear mixed model of PC 1 for shape including the fixed factor haplotype and the random factor of the mother snail´s ID in the F1 generation. Significance codes: 0 ‘***’ 0.001 ‘**’ 0.01 ‘*’ 0.05 ‘.’ 0.1 ‘ ’ 1.

|  | **Estimate** | **Standard error** | **df** | **t-value** | **p-value** |
| --- | --- | --- | --- | --- | --- |
| **Intercept** | 0.0064 | 0.0013 | 162.93 | 5.12 | 8.62e-07 *** |
| **Haplotype z** | -0.0150 | 0.0019 | 155.26 | -7.90 | 4.77e-13 *** |

**Supplemental Table S11.** Linear mixed model of PC 2 for shape including the fixed factors temperature, haplotype, population and the interaction of temperature and haplotype as well as the random factor of the mother snail´s ID in the F1 generation. Significance codes: 0 ‘***’ 0.001 ‘**’ 0.01 ‘*’ 0.05 ‘.’ 0.1 ‘ ’ 1.

|  | **Estimate** | **Standard error** | **df** | **t-value** | **p-value** |
| --- | --- | --- | --- | --- | --- |
| **Intercept** | -2.636e-02 | 8.314e-03 | 1.539e+02 | -3.17 | 0.001836 ** |
| **Temperature** | 1.022e-03 | 3.838e-04 | 1.521e+02 | 2.66 | 0.008577 ** |
| **Haplotype z** | -3.048e-02 | 1.232e-02 | 1.501e+02 | -2.48 | 0.014452 * |
| **Filzingen** | 1.106e-02 | 4.592e-03 | 1.497e+02 | 2.41 | 0.017281 * |
| **Hohensprenz** | 2.821e-03 | 4.663e-03 | 1.503e+02 | 0.61 | 0.546153 |
| **J008** | 1.100e-02 | 4.447e-03 | 1.442e+02 | 2.48 | 0.014497 * |
| **Jarmen** | 1.518e-02 | 5.031e-03 | 1.591e+02 | 3.02 | 0.002971 ** |
| **Kammlach** | 1.170e-02 | 4.649e-03 | 1.476e+02 | 2.52 | 0.012906 * |
| **Memmingen** | -3.590e-03 | 4.962e-03 | 1.516e+02 | -0.72 | 0.470479 |
| **Nascente do Sr. Jordão** | 9.441e-03 | 4.182e-03 | 1.479e+02 | 2.26 | 0.025428 * |
| **Porto de Mós** | 1.365e-02 | 3.915e-03 | 1.415e+02 | 3.49 | 0.000654 *** |
| **Póvoas village** | 7.617e-03 | 3.922e-03 | 1.424e+02 | 1.94 | 0.054074 . |
| **Temperature x Haplotype z** | 1.447e-03 | 5.881e-04 | 1.493e+02 | 2.46 | 0.015024 * |

**Supplemental Table S12.** Linear mixed model of PC 3 for shape including the fixed factors temperature and haplotype and the random factor of the mother snail´s ID in the F1 generation. Significance codes: 0 ‘***’ 0.001 ‘**’ 0.01 ‘*’ 0.05 ‘.’ 0.1 ‘ ’ 1.

|  | **Estimate** | **Standard error** | **df** | **t-value** | **p-value** |
| --- | --- | --- | --- | --- | --- |
| **Intercept** | 2.736e-02 | 4.420e-03 | 1.586e+02 | 6.191 | 4.92e-09 *** |
| **Temperature** | -1.060e-03 | 2.206e-04 | 1.544e+02 | -4.804 | 3.66e-06 *** |
| **Haplotype z** | -1.423e-02 | 1.440e-03 | 1.522e+02 | -9.878 | < 2e-16 *** |

**Appendix 5: Comparison of reaction norm slopes**

**Supplemental Table S13.** Comparison of reaction norm slopes of length across populations of the F1 generation using ANOVA and the calculation of the least square means. Significant differences (p < 0.05) are written in bold.


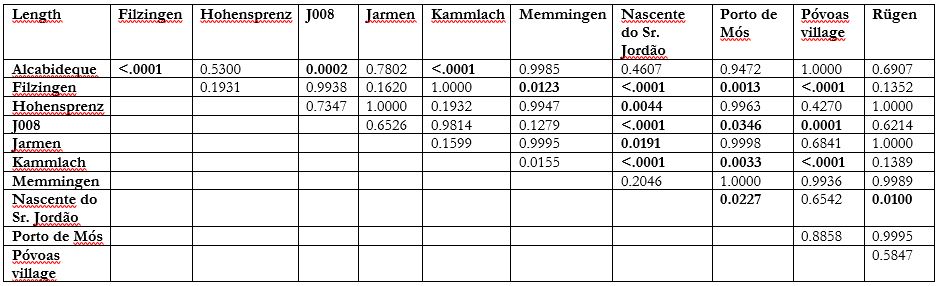


**Supplemental Table S14.** Comparison of reaction norm slopes of PC 1 for shape across populations of the F1 generation using ANOVA and the calculation of the least square means. Significant differences (p < 0.05) are written in bold.


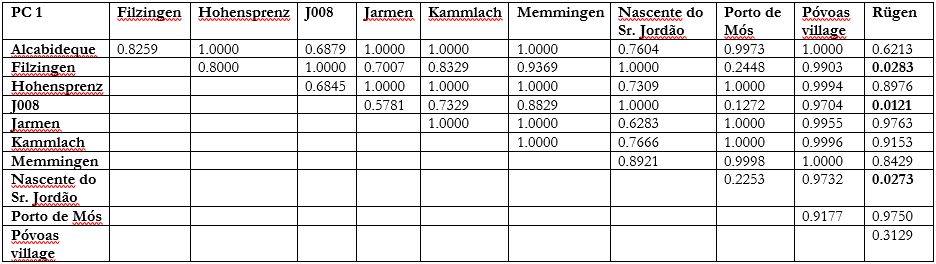


**Supplemental Table S15.** Comparison of reaction norm slopes of PC 2 for shape across populations of the F1 generation using ANOVA and the calculation of the least square means. Significant differences (p < 0.05) are written in bold.

**
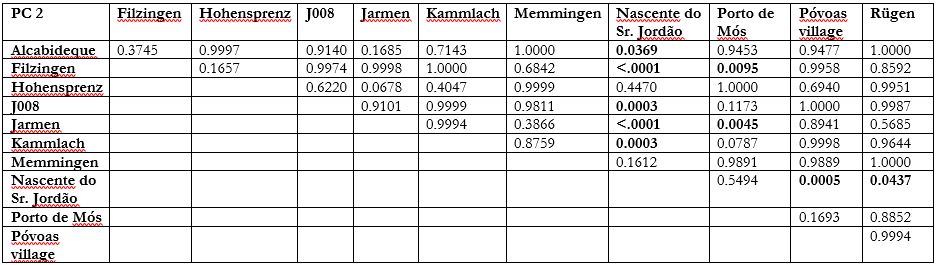
**

**Supplemental Table S16.** Comparison of reaction norm slopes of PC 3 for shape across populations of the F1 generation using ANOVA and the calculation of the least square means. Significant differences (p < 0.05) are written in bold.


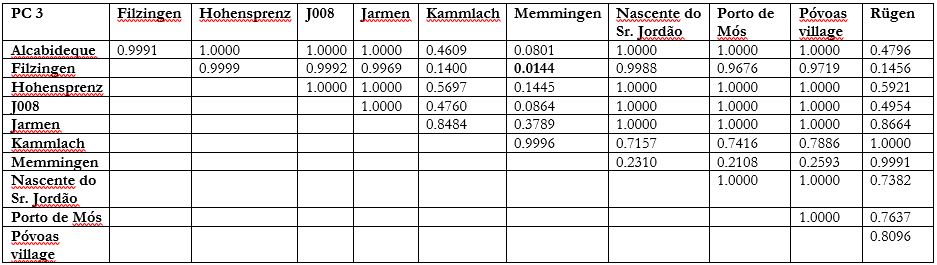


**Appendix 6: Haplotype-specific heritabilities**

**Supplemental Table S17.** Genetic variance in the F1 generation at 15°C. Overall genetic variance (*V_A_*), residual variance (*V_res_*), and broad-sense heritability (*H^2^*) are given for all size and shape parameters, additionally, coefficient of genetic variation (*CV_A_*) and its square (*I_A_*) for the size parameters. Significant *p* values are written in bold.

| **15°C** | ***V_A_*** | ***V_res_*** | ***H^2^*** | ***CV_A_*** | ***I_A_*** | ***p*** |
| --- | --- | --- | --- | --- | --- | --- |
| **Length** | 88032.95 | 47518.18 | 0.65 | 0.0066 | 4.37e-09 | **< 0.01** |
| **Centroid size** | 148960.2 | 158173.8 | 0.49 | 0.0039 | 1.49e-09 | **< 0.01** |
| **PC 1** | 0.000161 | 0.000225 | 0.42 | - | - | **< 0.01** |
| **PC 2** | 0.000223 | 0.000174 | 0.56 | - | - | **< 0.01** |
| **PC 3** | 0.000121 | 0.000139 | 0.47 | - | - | **< 0.01** |

**Supplemental Table S18.** Genetic variance in the F1 generation at 19°C. Overall genetic variance (*V_A_*), residual variance (*V_res_*), and broad-sense heritability (*H^2^*) are given for all size and shape parameters, additionally, coefficient of genetic variation (*CV_A_*) and its square (*I_A_*) for the size parameters. Significant *p* values are written in bold.

| **19°C** | ***V_A_*** | ***V_res_*** | ***H^2^*** | ***CV_A_*** | ***I_A_*** | ***p*** |
| --- | --- | --- | --- | --- | --- | --- |
| **Length** | 141414.41 | 34722.71 | 0.80 | 0.0106 | 1.13e-08 | **< 0.01** |
| **Centroid size** | 254133.2 | 124710.2 | 0.67 | 0.0066 | 4.33e-09 | **< 0.01** |
| **PC 1** | 0.00012 | 0.000322 | 0.27 | - | - | **< 0.01** |
| **PC 2** | 0.000133 | 0.000212 | 0.39 | - | - | **< 0.01** |
| **PC 3** | 0.000075 | 0.000172 | 0.30 | - | - | **< 0.01** |

**Supplemental Table S19.** Genetic variance in the F1 generation at 23°C. Overall genetic variance (*V_A_*), residual variance (*V_res_*), and broad-sense heritability (*H^2^*) are given for all size and shape parameters, additionally, coefficient of genetic variation (*CV_A_*) and its square (*I_A_*) for the size parameters. Significant *p* values are written in bold.

| **23°C** | ***V_A_*** | ***V_res_*** | ***H^2^*** | ***CV_A_*** | ***I_A_*** | ***p*** |
| --- | --- | --- | --- | --- | --- | --- |
| **Length** | 150940.89 | 41638.51 | 0.78 | 0.0113 | 1.29e-08 | **< 0.01** |
| **Centroid size** | 233094 | 113647.5 | 0.67 | 0.0060 | 3.64e-09 | **< 0.01** |
| **PC 1** | 0.000168 | 0.00033 | 0.34 | - | - | **< 0.01** |
| **PC 2** | 0.000117 | 0.00016 | 0.42 | - | - | **< 0.01** |
| **PC 3** | 0.000129 | 0.000148 | 0.47 | - | - | **< 0.01** |

**Supplemental Table S20.** Genetic variance in haplotype t of the F1 generation at 15°C. Overall genetic variance (*V_A_*), residual variance (*V_res_*), and broad-sense heritability (*H^2^*) are given for all size and shape parameters, additionally, coefficient of genetic variation (*CV_A_*) and its square (*I_A_*) for the size parameters. Significant *p* values are written in bold.

| **Haplotype t, 15°C** | | | | | | |
| --- | --- | --- | --- | --- | --- | --- |
|  | ***V_A_*** | ***V_res_*** | ***H_2_*** | ***CV_A_*** | ***I_A_*** | ***p*** |
| **Length** | 58477.77 | 56286.2 | 0.51 | 0.0038 | 1.47e-09 | **< 0.01** |
| **Centroid size** | 86618.51 | 172421.92 | 0.33 | 0.0020 | 4.09e-10 | **< 0.01** |
| **PC 1** | 0.000065 | 0.000194 | 0.25 | - | - | **< 0.01** |
| **PC 2** | 0.000247 | 0.000155 | 0.61 | - | - | **< 0.01** |
| **PC 3** | 0.000052 | 0.000149 | 0.26 | - | - | **< 0.01** |

**Supplemental Table S21.** Genetic variance in haplotype t of the F1 generation at 19°C. Overall genetic variance (*V_A_*), residual variance (*V_res_*), and broad-sense heritability (*H^2^*) are given for all size and shape parameters, additionally, coefficient of genetic variation (*CV_A_*) and its square (*I_A_*) for the size parameters. Significant *p* values are written in bold.

| **Haplotype t, 19°C** | | | | | | |
| --- | --- | --- | --- | --- | --- | --- |
|  | ***V_A_*** | ***V_res_*** | ***H_2_*** | ***CV_A_*** | ***I_A_*** | ***p*** |
| **Length** | 60845.55 | 41509.81 | 0.59 | 0.004 | 1.60e-09 | **< 0.01** |
| **Centroid size** | 101464.7 | 119119.5 | 0.46 | 0.0024 | 5.61e-10 | **< 0.01** |
| **PC 1** | 0.000083 | 0.000298 | 0.22 | - | - | **< 0.01** |
| **PC 2** | 0.000148 | 0.000174 | 0.46 | - | - | **< 0.01** |
| **PC 3** | 0.000051 | 0.00018 | 0.22 | - | - | **< 0.01** |

**Supplemental Table S22.** Genetic variance in haplotype t of the F1 generation at 23°C. Overall genetic variance (*V_A_*), residual variance (*V_res_*), and broad-sense heritability (*H^2^*) are given for all size and shape parameters, additionally, coefficient of genetic variation (*CV_A_*) and its square (*I_A_*) for the size parameters. Significant *p* values are written in bold.

| **Haplotype t, 23°C** | | | | | | |
| --- | --- | --- | --- | --- | --- | --- |
|  | ***V_A_*** | ***V_res_*** | ***H_2_*** | ***CV_A_*** | ***I_A_*** | ***p*** |
| **Length** | 25144.97 | 45184.48 | 0.36 | 0.0017 | 2.73e-10 | **< 0.01** |
| **Centroid size** | 45672.2 | 112898.1 | 0.29 | 0.0011 | 1.14e-10 | **< 0.01** |
| **PC 1** | 0.000067 | 0.000297 | 0.18 | - | - | **< 0.01** |
| **PC 2** | 0.00009 | 0.000142 | 0.39 | - | - | **< 0.01** |
| **PC 3** | 0.000069 | 0.000148 | 0.32 | - | - | **< 0.01** |

**Supplemental Table S23.** Genetic variance in haplotype z of the F1 generation at 15°C. Overall genetic variance (*V_A_*), residual variance (*V_res_*), and broad-sense heritability (*H^2^*) are given for all size and shape parameters, additionally, coefficient of genetic variation (*CV_A_*) and its square (*I_A_*) for the size parameters. Significant *p* values are written in bold.

| **Haplotype z, 15°C** | | | | | | |
| --- | --- | --- | --- | --- | --- | --- |
|  | ***V_A_*** | ***V_res_*** | ***H_2_*** | ***CV_A_*** | ***I_A_*** | ***p*** |
| **Length** | 21593.71 | 38882.8 | 0.36 | 0.002 | 3.86e-10 | **< 0.01** |
| **Centroid size** | 45368.73 | 142690.28 | 0.24 | 0.0014 | 1.84e-10 | **< 0.01** |
| **PC 1** | 0.000108 | 0.000252 | 0.30 | - | - | **< 0.01** |
| **PC 2** | 0.000179 | 0.000191 | 0.48 | - | - | **< 0.01** |
| **PC 3** | 0.000034 | 0.000130 | 0.21 | - | - | **< 0.01** |

**Supplemental Table S24.** Genetic variance in haplotype z of the F1 generation at 19°C. Overall genetic variance (*V_A_*), residual variance (*V_res_*), and broad-sense heritability (*H^2^*) are given for all size and shape parameters, additionally, coefficient of genetic variation (*CV_A_*) and its square (*I_A_*) for the size parameters. Significant *p* values are written in bold.

| **Haplotype z, 19°C** | | | | | | |
| --- | --- | --- | --- | --- | --- | --- |
|  | ***V_A_*** | ***V_res_*** | ***H_2_*** | ***CV_A_*** | ***I_A_*** | ***p*** |
| **Length** | 15601.36 | 26220.15 | 0.37 | 0.0014 | 2.01e-10 | **< 0.01** |
| **Centroid size** | 41061.25 | 130756.52 | 0.24 | 0.0012 | 1.51e-10 | **< 0.01** |
| **PC 1** | 0.000074 | 0.000346 | 0.18 | - | - | **< 0.01** |
| **PC 2** | 0.000111 | 0.000248 | 0.31 | - | - | **< 0.01** |
| **PC 3** | 0.000039 | 0.000165 | 0.19 | - | - | **< 0.01** |

**Supplemental Table S25.** Genetic variance in haplotype z of the F1 generation at 23°C. Overall genetic variance (*V_A_*), residual variance (*V_res_*), and broad-sense heritability (*H^2^*) are given for all size and shape parameters, additionally, coefficient of genetic variation (*CV_A_*) and its square (*I_A_*) for the size parameters. Significant *p* values are written in bold.

| **Haplotype z, 23°C** | | | | | | |
| --- | --- | --- | --- | --- | --- | --- |
|  | ***V_A_*** | ***V_res_*** | ***H_2_*** | ***CV_A_*** | ***I_A_*** | ***p*** |
| **Length** | 18380.78 | 35619.87 | 0.34 | 0.0017 | 2.79e-10 | **< 0.01** |
| **Centroid size** | 67846.27 | 115495.07 | 0.37 | 0.0020 | 4.12e-10 | **< 0.01** |
| **PC 1** | 0.000206 | 0.000389 | 0.35 | - | - | **< 0.01** |
| **PC 2** | 0.000159 | 0.000189 | 0.46 | - | - | **< 0.01** |
| **PC 3** | 0.000101 | 0.000148 | 0.40 | - | - | **< 0.01** |

**Appendix 7: Morphological traits in the F2 generation**

As the F1 generation did not produce enough offspring in all populations within the scheduled time, we could analyse only two Portuguese populations, Alcabideque and Póvoas village, both with haplotype z, across all three generations. The second offspring generation F2 had a sample size of n = 318 with the shape analyses and n = 300 with the size analyses. Length and centroid size were significantly different compared to the parent generation and the F1 generation [Kruskal-Wallis chi-squared = 68.81, df = 2, p < 0.01 (length); Kruskal-Wallis chi-squared = 18.16, df = 2, p < 0.01 (centroid size)] (Supplemental Figures S3 and S4). There was no significant difference in length between F1 and F2 from Póvoas village. In centroid size, we only found a significant difference for Alcabideque between the parental and the F1 generation and between F1 and F2 generations. The model with the interaction of temperature and population as fixed factor and the mother snail´s ID as random factor had the lowest AIC for both, length and centroid size (Supplemental Tables S25 and S26). For length, the marginal *R^2^* was 0.47 and the conditional *R^2^* was 0.65. The random factor therefore explained 18% of the variance in the model. For centroid size, the marginal *R^2^* was 0.32 and the conditional *R^2^* was 0.63, the random factor thus accounted for 31% of the variance.

For all shape parameters, there was a significant difference between the three generations [Kruskal-Wallis chi-squared = 65.32, df = 2, p < 0.01 (PC 1); Kruskal-Wallis chi-squared = 36.87, df = 2, p < 0.01 (PC 2); Kruskal-Wallis chi-squared = 37.49, df = 2, p < 0.01 (PC 3)] – with some exceptions (Supplemental Figure S5). For PC 1, snails from Alcabideque did not show any significant differences between the F1 and the F2 generations and those from Póvoas village did not show any differences between the parental and the F1 generations. We did not see any significance in PC 2 between the parental and the F2 generations in Alcabideque and between the F1 and the F2 generations in Póvoas village. Lastly, PC 3 did not show a significant difference between the F1 and the F2 generations in both Alcabideque and Póvoas village. The best model for PC 1 contained the fixed factors temperature and population, for PC 2 temperature and for PC 3 the interaction of temperature and population (Supplemental Tables S27-S29). The marginal and the conditional *R^2^* for PCs 1, 2 and 3 were 0.17 and 0.33, 0.40 and 0.54, as well as 0.13 and 0.25, respectively. The random effect maternal ID described 16% of the variation in PC 1, 14% in PC 2, and 12% in PC 3.


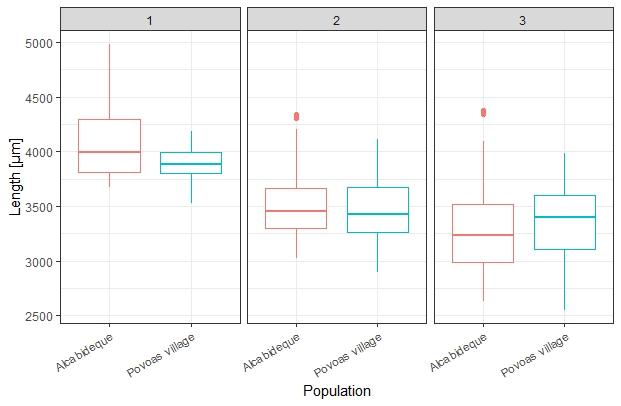


**Supplemental Figure S3.** Length across the three generations in the populations Alcabideque and Póvoas village.


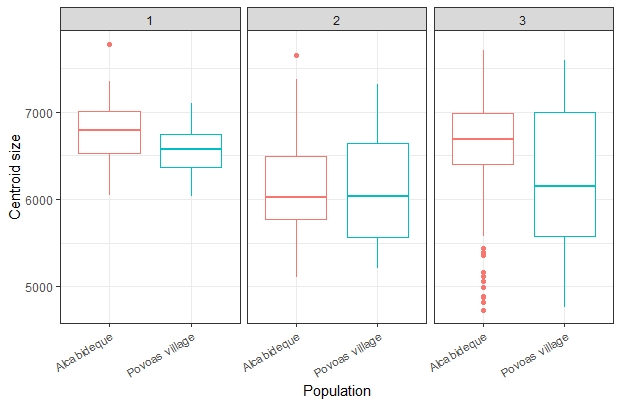


**Supplemental Figure S4.** Centroid size across the three generations in the populations Alcabideque and Póvoas village.


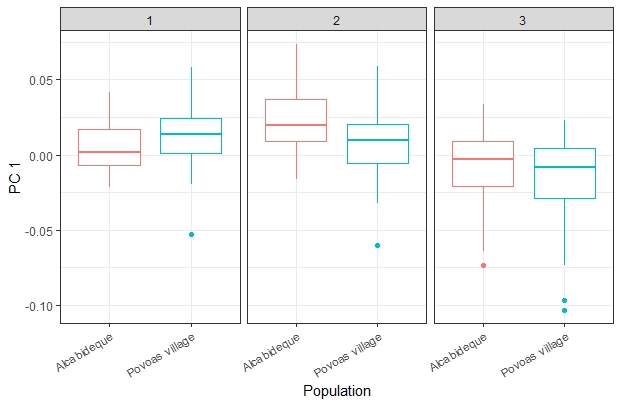


**
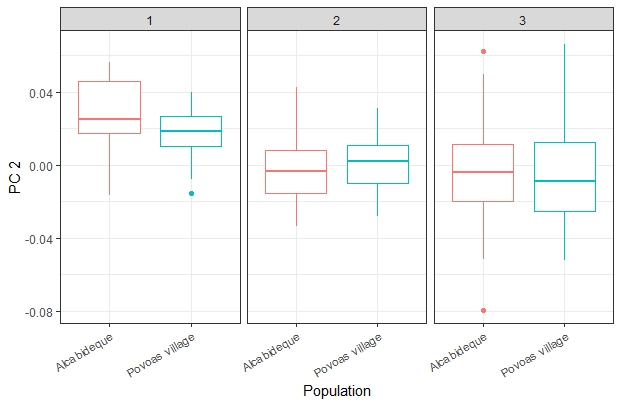
**

**
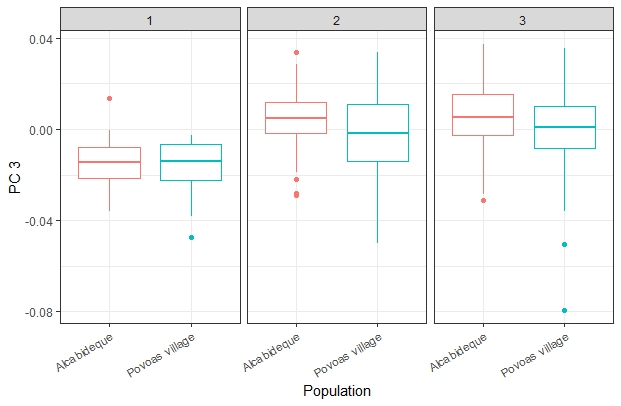
**

**Supplemental Figure S5.** PC 1-3 for shape across the three generations (1-3) in the populations Alcabideque and Póvoas village.

**Supplemental Table S26.** Linear mixed model of length including the fixed factor, the interaction of temperature and population, and the random factor of the mother snail´s ID in the F2 generation. Significance codes: 0 ‘***’ 0.001 ‘**’ 0.01 ‘*’ 0.05 ‘.’ 0.1 ‘ ’ 1.

|  | **Estimate** | **Standard error** | **df** | **t-value** | **p-value** |
| --- | --- | --- | --- | --- | --- |
| **Intercept** | 3998.42 | 102.54 | 34.04 | 38.995 | < 2e-16 *** |
| **Temperature 19°C** | -478.26 | 130.75 | 35.34 | -3.658 | 0.0008 *** |
| **Temperature 23°C** | -921.66 | 117.50 | 31.53 | -7.844 | 6.64e-09 *** |
| **Population Póvoas village** | -432.96 | 122.38 | 34.10 | -3.538 | 0.0012 ** |
| **Temperature 19°C x Population Póvoas village** | 457.38 | 237.73 | 40.28 | 1.924 | 0.0614 . |
| **Temperature 23°C x Population Póvoas village** | 520.45 | 149.55 | 31.64 | 3.480 | 0.0015 ** |

**Supplemental Table S27.** Linear mixed model of centroid size including the fixed factor, the interaction of temperature and population, and the random factor of the mother snail´s ID in the F2 generation. Significance codes: 0 ‘***’ 0.001 ‘**’ 0.01 ‘*’ 0.05 ‘.’ 0.1 ‘ ’ 1.

|  | **Estimate** | **Standard error** | **df** | **t-value** | **p-value** |
| --- | --- | --- | --- | --- | --- |
| **Intercept** | 7344.55 | 187.48 | 39.22 | 39.174 | < 2e-16 *** |
| **Temperature 19°C** | -387.93 | 238.82 | 40.48 | -1.624 | 0.112 |
| **Temperature 23°C** | -985.87 | 215.30 | 36.65 | -4.579 | 5.23e-05 *** |
| **Population Póvoas village** | -315.69 | 223.76 | 39.24 | -1.411 | 0.166 |
| **Temperature 19°C x Population Póvoas village** | 348.24 | 432.76 | 45.08 | 0.805 | 0.425 |
| **Temperature 23°C x Population Póvoas village** | -502.16 | 274.02 | 36.71 | -1.833 | 0.075 . |

**Supplemental Table S28.** Linear mixed model of PC 1 for shape including the fixed factors of temperature and population and the random factor of the mother snail´s ID in the F2 generation. Significance codes: 0 ‘***’ 0.001 ‘**’ 0.01 ‘*’ 0.05 ‘.’ 0.1 ‘ ’ 1.

|  | **Estimate** | **Standard error** | **df** | **t-value** | **p-value** |
| --- | --- | --- | --- | --- | --- |
| **Intercept** | 0.006646 | 0.005431 | 37.63 | 1.224 | 0.22865 |
| **Temperature 19°C** | -0.002970 | 0.007105 | 43.81 | -0.418 | 0.67798 |
| **Temperature 23°C** | -0.019493 | 0.005452 | 36.25 | -3.575 | 0.00101 ** |
| **Population Póvoas village** | -0.010570 | 0.004793 | 34.48 | -2.205 | 0.03420 * |

**Supplemental Table S29.** Linear mixed model of PC 2 for shape including the fixed factor temperature and the random factor of the mother snail´s ID in the F2 generation. Significance codes: 0 ‘***’ 0.001 ‘**’ 0.01 ‘*’ 0.05 ‘.’ 0.1 ‘ ’ 1.

|  | **Estimate** | **Standard error** | **df** | **t-value** | **p-value** |
| --- | --- | --- | --- | --- | --- |
| **Intercept** | -0.026731 | 0.004243 | 36.90 | -6.300 | 2.49e-07 *** |
| **Temperature 19°C** | 0.005941 | 0.006755 | 40.38 | 0.879 | 0.384 |
| **Temperature 23°C** | 0.036178 | 0.005291 | 32.81 | 6.838 | 8.64e-08 *** |

**Supplemental Table S30.** Linear mixed model of PC 3 for shape including the fixed factor, the interaction of temperature and population, and the random factor of the mother snail´s ID in the F2 generation. Significance codes: 0 ‘***’ 0.001 ‘**’ 0.01 ‘*’ 0.05 ‘.’ 0.1 ‘ ’ 1.

|  | **Estimate** | **Standard error** | **df** | **t-value** | **p-value** |
| --- | --- | --- | --- | --- | --- |
| **Intercept** | -0.004126 | 0.005113 | 41.72 | -0.807 | 0.42419 |
| **Temperature 19°C** | 0.004699 | 0.006578 | 44.72 | 0.714 | 0.47879 |
| **Temperature 23°C** | 0.012300 | 0.005761 | 37.13 | 2.135 | 0.03940 * |
| **Population Póvoas village** | 0.008110 | 0.006109 | 42.30 | 1.327 | 0.19149 |
| **Temperature 19°C x Population Póvoas village** | -0.006041 | 0.009958 | 50.77 | -0.607 | 0.54683 |
| **Temperature 23°C x Population Póvoas village** | -0.022871 | 0.007345 | 37.75 | -3.114 | 0.00351 ** |

**Appendix 8: Shape comparison of parental and F1 generations**

The snails of the F1 generation that were born and raised in the common garden showed higher scores on PCs 1 and 2 and lower ones along PC 3 than their mothers. In comparison to the mother snails, the F1 generation possesses more slender shells with a relatively smaller body whorl (Supplemental Figure S6).


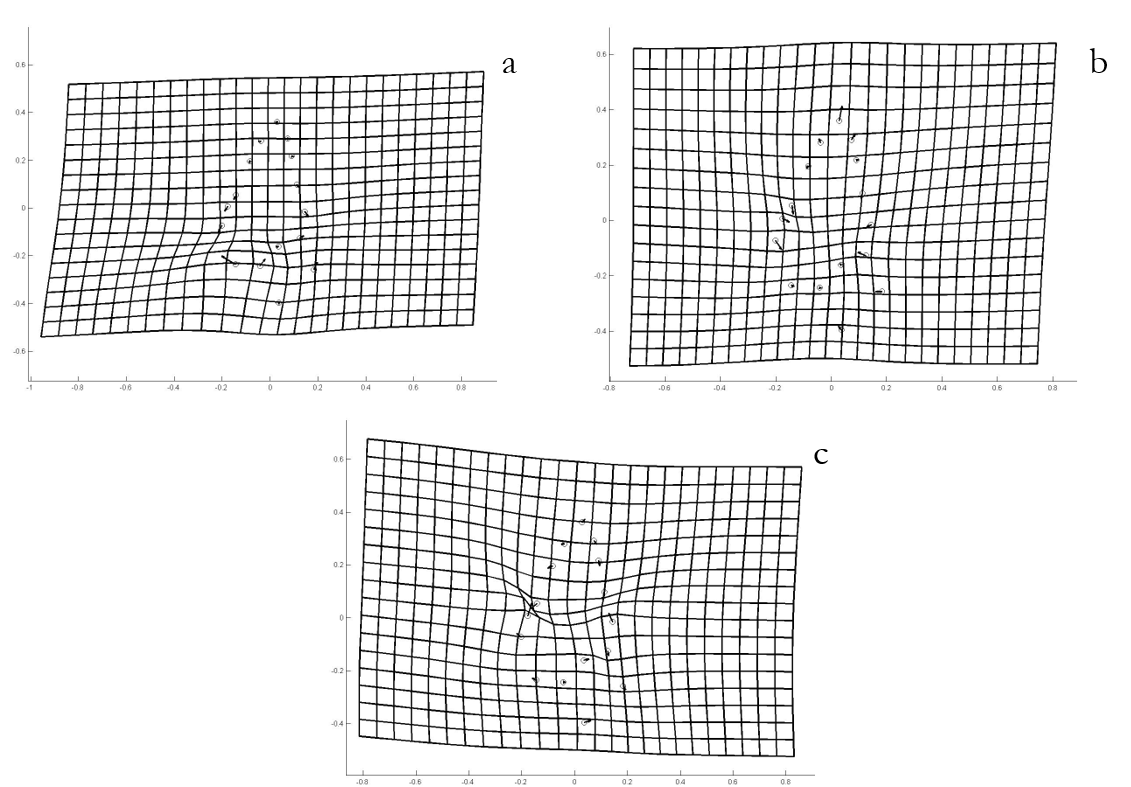


**Supplemental Figure S6.** Deformation grids for PC 1 (a), PC 2 (b) and PC 3 (c) of the parental and the F1 generation. Grids show deformation from specimens with lowest (circle) to those with highest scores (arrowhead).

**Appendix 9: Life history**


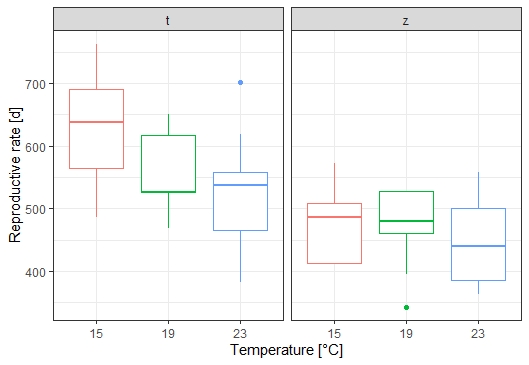


**Supplemental Figure S7.** Reproductive rate of the parental generation depending on climate cabinet temperature and haplotype. Only the mother snails with haplotype t of the 15°C and the 23 °C climate cabinets differed significantly.

**Supplemental Table S31**. Generalized linear model of the reproductive rate including the fixed factor, the interaction of temperature and haplotype in the parental generation. Significance codes: 0 ‘***’ 0.001 ‘**’ 0.01 ‘*’ 0.05 ‘.’ 0.1 ‘ ’ 1.

|  | **Estimate** | **Standard error** | **t-value** | **p-value** |
| --- | --- | --- | --- | --- |
| **Intercept** | 861.827 | 67.374 | 12.792 | < 2e-16 *** |
| **Temperature** | -14.834 | 3.336 | -4.447 | 3.05e-05 *** |
| **Haplotype z** | -321.039 | 93.939 | -3.418 | 0.00104 ** |
| **Temperature x Haplotype z** | 10.988 | 4.759 | 2.309 | 0.02378 * |
